# Genome-wide characterization of tea plant (*Camellia sinensis*) Hsf transcription factor family and role of *CsHsfA2* in heat tolerance

**DOI:** 10.1186/s12870-020-02462-9

**Published:** 2020-05-29

**Authors:** Xuyang Zhang, Wenluan Xu, Dejiang Ni, Mingle Wang, Guiyi Guo

**Affiliations:** 1 Key Laboratory of Horticultural Plant Biology (Ministry of Education), College of Horticulture and Forestry Sciences, Huazhong Agricultural University, Shizishan No. 1, Wuhan, 430070 Hubei Province P. R. China; 2 Henan Key Laboratory of Tea Plant Comprehensive Utilization in South Henan, Xinyang Agriculture and Forestry University, Xinyang, 464000 China

**Keywords:** Hsf family, Abiotic stress, Expression patterns, *CsHsfA2*, Thermotolerance

## Abstract

**Background:**

Heat stress factors (Hsfs) play vital roles in signal transduction pathways operating in responses to environmental stresses. However, *Hsf* gene family has not been thoroughly explored in tea plant (*Camellia sinensis* L.).

**Results:**

In this study, we identified 25 *CsHsf* genes in *C. sinensis* that were separated by phylogenetic analysis into three sub-families (i.e., A, B, and C). Gene structures, conserved domains and motifs analyses indicated that the CsHsf members in each class were relatively conserved. Various *cis*-acting elements involved in plant growth regulation, hormone responses, stress responses, and light responses were located in the promoter regions of *CsHsfs*. Furthermore, degradome sequencing analysis revealed that 7 *CsHsfs* could be targeted by 9 miRNAs. The expression pattern of each *CsHsf* gene was significantly different in eight tissues. Many *CsHsfs* were differentially regulated by drought, salt, and heat stresses, as well as exogenous abscisic acid (ABA) and Ca^2+^. In addition, CsHsfA2 was located in the nucleus. Heterologous expression of *CsHsfA2* improved thermotolerance in transgenic yeast, suggesting its potential role in the regulation of heat stress response.

**Conclusions:**

A comprehensive genome-wide analysis of Hsf in *C. sinensis* present the global identification and functional prediction of *CsHsfs*. Most of them were implicated in a complex gene regulatory network controlling various abiotic stress responses and signal transduction pathways in tea plants. Additionally, heterologous expression of *CsHsfA2* increased thermotolerance of transgenic yeast. These findings provide new insights into the functional divergence of *CsHsfs* and a basis for further research on CsHsfs functions.

## Background

Over the years, with the release of the genomic sequences of two primary tea plant varieties: *Camellia sinensis var. sinensis* and *Camellia sinensis var. assamica* [1], several transcription factors families have been identified, such as lateral organ boundaries (LBD) [2], homeodomain-leucine zipper (HD-Zip) [3], gibberellic acid insensitive (GAI), repressor of GA1 (RGA), scarecrow (SCR) (GRAS) [4], SQUAMOSA promoter-binding protein-like (SPL) [5, 6], nuclear factor-Y (NF-Y) [7], and CCAAT-binding factor (CBF) [8]. Hsfs are one of the largest transcription factors families in plant genomes and form integral parts of signalling webs that modulate many biological processes, especially heat stress (HS) [9, 10]. They can specifically recognize the HS elements (HSE; 5′-AGAAnnTTCT-3′) conserved in promoters of HS-inducible genes [11]. Although numerous Hsf family numbers have been identified from Chinese white pear (*Pyrus bretschneideri*) [12], Chinese cabbage (*Brassica rapa*) [13], *Brassica oleracea* [14], *Triticum aestivum* [15], *Salix suchowensis* [16], and five model angiosperms (*Arabidopsis thaliana*, *Cucumis sativus*, *Oryza sativa*, *Populus trichocarpa*, and *Vitis vinifera*) [17, 18], no systematic identification of *Hsf* family is available in tea plant. The elucidation of *CsHsfs* function and regulation in tea plant will provide foundation for further functional research of *Hsf* genes in evergreen woody crops.

Hsf transcription factors play vital roles in the regulation of plant response to abiotic stresses, such as salinity, drought, osmotic stress, cold, and HS [9, 19, 20]. For example, *HsfA2*, a typical representative of plant *Hsfs*, was a HS-inducible gene and regulated a subset of down-stream stress response genes [19]. Overexpression of *HsfA2* from *A. thaliana*, *Zea mays*, *Lilium longiflorum*, or *O. sativa*, conferred heat tolerance in transgenic Arabidopsis [21–24]. *GmHsfA1* overexpression also enhanced thermotolerance of transgenic soybean under HS [25]. The drought and heat stress induced expression of *HsfA3* in Arabidopsis depends on the dehydration-responsive element-binding protein 2A (DREB2A) [26, 27]. Although *O. sativa HsfB2b* expression was strongly induced by heat, salt, abscisic acid (ABA) and polyethylene glycol (PEG) treatments but not cold stress, *OsHsfB2b* overexpression increased the drought and salt sensitivity in rice [28]. However, *Hordeum vulgare HsfB2c* was proposed to be positively regulating the drought stress tolerance in barley [29], and *CmHSFA4* and *AtHSFA7b* positively regulated salt stress tolerance in chrysanthemum and Arabidopsis, respectively [30, 31]. Moreover, *OsHsfC1b* and *T. aestivum HsfC2a* overexpression improved salt tolerance and thermotolerance in transgenic *O. sativa* and wheat, respectively [32, 33]. In addition to its role in stress responses, it is interesting to note that overexpression of sunflower *HsfA9* improved seed longevity in transgenic tobacco [34]. Thus, the unique functions of specific *Hsf* from different plant species remain to be elucidated.

Tea plants [*Camellia sinensis* (L.) O. Kuntze] are one of the most important commercial perennial evergreen leaf crops used for the production of non-alcoholic beverages in china and worldwide [35]. Most of tea plant species are diploids with a genome size of thirty chromosomes [36]. Being sessile organisms, tea plants have evolved a series of complex mechanisms to cope with fluctuating environmental stresses, such as extreme temperatures [37], drought [38, 39], soil acidification [40, 41], and heavy metals [42, 43]. Long-term hot summers hinder tea plants growth and development, seriously affecting the yield and quality of spring tea in the next year [44]. Hence, it is necessary to investigate the thermotolerance mechanisms of tea plants, which may be useful for HS-resistant cultivation and breeding in the future. We previously have attempted to discover the key HS-responsive genes using a suppression subtractive hybridization approach [45]. However, due to the technical limitations of suppression subtractive hybridization and unavailability of tea plant genome at that time, only 12 differentially expressed heat-responsive genes were identified, including one *Hsf* gene [45]. Although 16 *CsHsfs* have been identified from *C. sinensis* RNA-seq data [46], little information on the genome-wide identification of *CsHsf* genes is available. Given the potential value of *Hsfs* in resistance to HS, it is necessary to identify *Hsf* genes in tea plant genome. In this study, we initiated the characterization of *C. sinensis Hsf* gene family and the expression patterns of putative *CsHsfs* in responses to a variety of stress treatments. We also examined the heat resistance function of *CsHsfA2* using a yeast heterologous expression system. These findings provide new insights into the functional divergence of *CsHsf* genes and a basis for genetic engineering breeding of plant species.

## Results

### Identification of *Hsf* gene family in *C. sinensis*

A total of 25 putative *Hsf* genes were identified from *C. sinensis* cultivar ‘Shuchazao’ genome. Members of the *CsHsf* gene family were subdivided into classes A, B, and C according to differences in the length of the flexible linkers between the A and B parts of the HR-A/B regions, including 15 *CsHsfA* genes, 9 *CsHsfB* genes, and 1 *CsHsfC* genes (Table 1). The length of the *CsHsfs* coding sequences ranged from 624 bp (*CsHsfA5a*) to 1998 bp (*CsHsfA4b*) with 207–665 amino acid residues. The molecular masses of CsHsfs varied greatly, ranging from 23.56 kDa (CsHsfA5a) to 74.98 kDa (CsHsfA4b), the computed theoretical isoelectric points ranged from 4.71 (CsHsfA9b) to 10.01 (CsHsfB3c), and the grand average of hydropathicity (GRAVY) was between − 1.01 (CsHsfB3c) and − 0.34 (CsHsfA9c). Additionally, subcellular localization predictions indicated that most of CsHsf proteins were predicted to be located in the nucleus, while CsHsfA5a and CsHsfA8 were targeted to cytoplasm and chloroplast, respectively. The detailed information is listed in Table 1.

**Table 1:** Sequence characteristics of *CsHsf* genes

Gene name	Genome ID	ORF (bp)	Amino acids (aa)	MW (kDa)	Theoretical isoelectric point	GRAVY	Subcellular localization prediction
*CsHsfA1a*	TEA029045	1527	508	56.46	5.13	−0.55	Nucleus
*CsHsfA1b*	TEA030860	1542	513	56.78	4.78	−0.62	Nucleus
*CsHsfA2*	TEA023633	1203	400	45.39	4.85	−0.57	Nucleus
*CsHsfA3*	TEA018554	1722	573	63.81	5.02	−0.51	Nucleus
*CsHsfA4a*	TEA024058	1470	489	55.56	5.00	−0.75	Nucleus
*CsHsfA4b*	TEA014681	1998	665	74.98	5.91	−0.58	Nucleus
*CsHsfA5a*	TEA022550	624	207	23.56	5.68	−0.22	Cytoplasm
*CsHsfA5b*	TEA006268	1440	479	53.66	5.76	−0.77	Nucleus
*CsHsfA6*	TEA008111	1023	340	38.81	5.07	−0.79	Nucleus
*CsHsfA7*	TEA005927	1059	352	40.55	5.37	−0.86	Nucleus
*CsHsfA8*	TEA015988	1167	388	44.26	4.72	−0.63	Chloroplast
*CsHsfA9a*	TEA021869	1266	421	47.11	5.16	−0.60	Nucleus
*CsHsfA9b*	TEA016625	1116	371	41.43	4.71	−0.51	Nucleus
*CsHsfA9c*	TEA014089	1401	466	51.34	4.49	−0.34	Nucleus
*CsHsfA9d*	TEA014078	996	331	37.20	5.14	−0.58	Nucleus
*CsHsfB1*	TEA013918	1569	522	57.83	5.16	−0.63	Nucleus
*CsHsfB2a*	TEA012764	945	314	35.08	5.37	−0.68	Nucleus
*CsHsfB2b*	TEA022795	1104	367	40.70	5.35	−0.60	Nucleus
*CsHsfB2c*	TEA008064	900	299	34.02	6.27	−0.87	Nucleus
*CsHsfB3a*	TEA013885	723	240	27.87	6.67	−0.72	Nucleus
*CsHsfB3b*	TEA000588	723	240	27.83	5.59	−0.86	Nucleus
*CsHsfB3c*	TEA010217	519	172	19.44	10.01	−1.01	Nucleus
*CsHsfB4a*	TEA005751	849	282	32.80	6.42	−0.73	Nucleus
*CsHsfB4b*	TEA031831	906	301	33.38	6.49	−0.52	Nucleus
*CsHsfC1*	TEA022299	966	321	36.25	5.35	−0.51	Nucleus

ExPASy [47] and WoLF PSORT [48] were used to analyze the physicochemical parameters and subcellular localization, respectively. GRAVY stands for grand average of hydropathicity

### Identification of conserved motifs and domains in CsHsfs

A total of 25 conserved motifs were identified from CsHsfs using MEME [49]. Motifs 1 and 3 were found in all of the CsHsfs, while motif 13 only existed in class A CsHsfs (Additional file 1: Figure S1). In addition, the number of motifs in the CsHsfBs and CsHsfCs was less than those in the CsHsfAs, especially in CsHsfB3c, which harbored only two motifs.

**Fig. 1:**
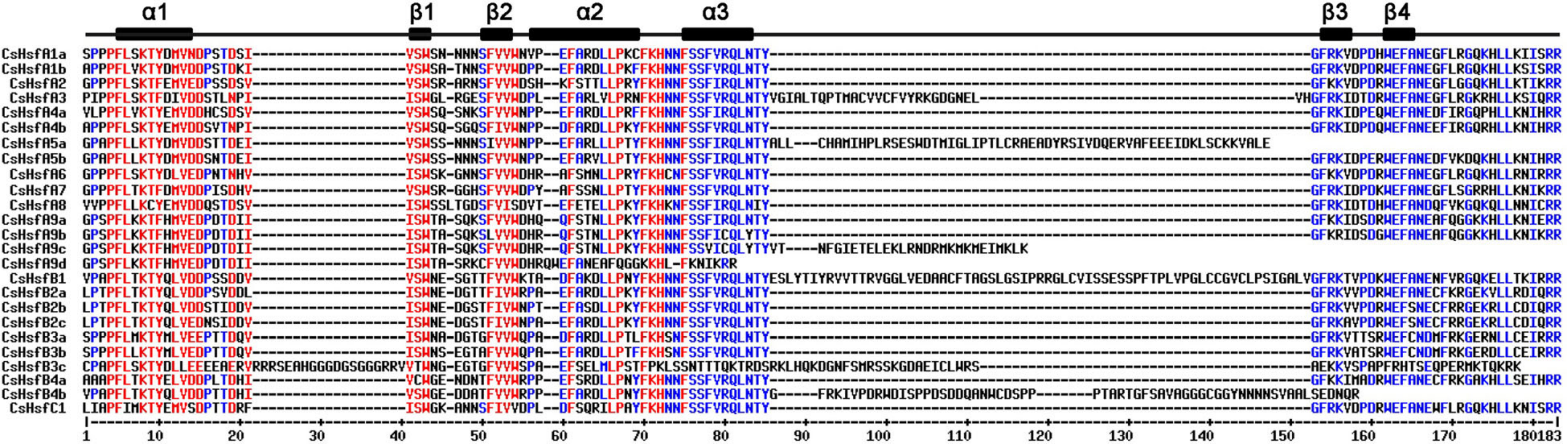
Multiple sequence alignment of DBD domains of CsHsf proteins. Three α-helices and four β-sheets were presented in the region. Sequences were aligned using MultAlin (version 5.4.1). Red and blue colors represent high (≥ 90%) and low (≤ 50%) sequence consensus, respectively

To better understand the structural characteristics of the CsHsf family, six conserved domains including DND-binding domain (DBD), oligomerization domain (HR-A/B), nuclear localization signal (NLS), nuclear export signal (NES), activator motifs (AHA), and repressor domain (RD) were identified using SMART [50], Pfam [51], NLStradamus [52] and NetNES [53] (Table 2). DBD is the most conserved domain, comprised of three α-helices and four β-sheets in the form of α1-β1-β2-α2-α3-β3-β4 (Fig. 1). However, no intact α3, β3, and β4 were detected in the DBD domain of CsHsfA5a, CsHsfA9c, CsHsfA9d, and CsHsfB4b, which may result in their shorter sequences compared to other CsHsfs. HR-A/B, which is characterized by a coiled-coil structure (coil-coil structure), was also present in most of the CsHsfs except for CsHsfA5a, CsHsfA5b, CsHsfA8, CsHsfA9c, CsHsfB3c, CsHsfB4a, and CsHsfB4b (Table 2). Moreover, NES and NLS domains were detected in most of the CsHsfs, which are vital for shuttling CsHsfs between the nucleus and cytoplasm. In addition, AHA and RD domains were specific to each class. AHA motifs were only detected in class A CsHsfs (except for CsHsfA5a and CsHsfA5b), and four proteins in subclasses A2, A3, and A4 had two AHA motifs. The tetrapeptide motif LFGV, as the core of the RD, was identified in all CsHsfB members except for CsHsfB3c. Interestingly, only one DBD domain was identified in CsHsfA5a and CsHsfB3c.

**Table 2:** Functional domains of CsHsfs

Gene name	Genome ID	DBD	HR-A/B	NLS	NES	AHA	RD
*CsHsfA1a*	TEA029045	21–114	138–178	(226)KKR	N.D.	(442)DVFWEQFLSV	N.D.
*CsHsfA1b*	TEA030860	31–124	153–205	(236)EGNKKRRLK	N.D.	(451)DPFWEQFLAS	N.D.
*CsHsfA2*	TEA023633	37–130	150–202	(225)KRKQRGVEIGRKRR	N.D.	(272)LFSAALDNE; (333)LEVDVEVEDL	N.D.
*CsHsfA3*	TEA018554	111–232	246–303	N.D.	N.D.	(410)IVSEELHVTH; (506)VLWDIVNNYD	N.D.
*CsHsfA4a*	TEA024058	93–186	207–235	N.D.	N.D.	(342)LMFWENIIHD; (427)DVFWEQFLTE	N.D.
*CsHsfA4b*	TEA014681	10–103	118–166	N.D.	(242)LIEKLESSL	(249)LNFWENFIRG; (379)DVFWEQFLTE	N.D.
*CsHsfA5a*	TEA022550	8–129	N.D.	N.D.	N.D.	N.D.	N.D.
*CsHsfA5b*	TEA006268	8–101	N.D.	N.D.	(136)LEKAACEKAALLSAAKLQL	N.D.	N.D.
*CsHsfA6*	TEA008111	29–122	146–187	(119)IRRRK	(269)LEQL	(305)EGFWEDLLNE	N.D.
*CsHsfA7*	TEA005927	36–129	211–242	(119)RRHLLKNIKRRKVP	N.D.	(307)ERFWEELLNE	N.D.
*CsHsfA8*	TEA015988	11–105	N.D.	N.D.	(283)LRDFFM	(318)DSILEQLLLS	N.D.
*CsHsfA9a*	TEA021869	93–186	208–240	N.D.	(219)NDRMKM	(358)LWEKLLED	N.D.
*CsHsfA9b*	TEA016625	95–188	205–242	(177)GKKHLLKNIKRRKHN	N.D.	(317)YILWEKLLED	N.D.
*CsHsfA9c*	TEA014089	222–312	N.D.	N.D.	(299)NDRM	(403)YILWEKLLED	N.D.
*CsHsfA9d*	TEA014078	72–131	153–188	(120)GKKHLFKNIKRRKH	(164)NDRMKM	(268)YILWEKLLED	N.D.
*CsHsfB1*	TEA013918	6–166	217–254	(162)KIRRRKAL	N.D.	N.D.	(303)LFGV
*CsHsfB2a*	TEA012764	21–114	178–211	N.D.	N.D.	N.D.	(253)LFGV
*CsHsfB2b*	TEA022795	26–119	173–208	N.D.	(284)KELRLQL	N.D.	(261)LFGV
*CsHsfB2c*	TEA008064	22–115	154–191	N.D.	(172)MQL	N.D.	(235)LFGV
*CsHsfB3a*	TEA013885	26–119	153–186	N.D.	(194)LKEEEEDDERPKLFGVRL	N.D.	(206)LFGV
*CsHsfB3b*	TEA000588	18–111	148–176	(220)RKRKR	N.D.	N.D.	(206)LFGV
*CsHsfB3c*	TEA010217	27–160	N.D.	N.D.	N.D.	N.D.	N.D.
*CsHsfB4a*	TEA005751	39–132	N.D.	(7)RRRRRR	N.D.	N.D.	(272)LFGV
*CsHsfB4b*	TEA031831	21–283	N.D.	N.D.	(286)LEKDDLGLNL	N.D.	(257)LFGV
*CsHsfC1*	TEA022299	6–99	111–148	(90)KHLLKNISRRKHNKQRK	(171)LDKEKKRRLLI	N.D.	N.D.

SMART [50], Pfam [51], NLStradamus [52], and NetNES [53] were used to identify these conserved domains, respectively. *DBD* DND-binding domain, *HR-A/B* oligomerization domain, *NLS* nuclear localization signal, *NES* nuclear export signal, *AHA* activator motifs, *RD* repressor domain, *N.D.* no motifs detectable by sequence similarity search

### Phylogenetic analysis of CsHsfs

**Fig. 2:**
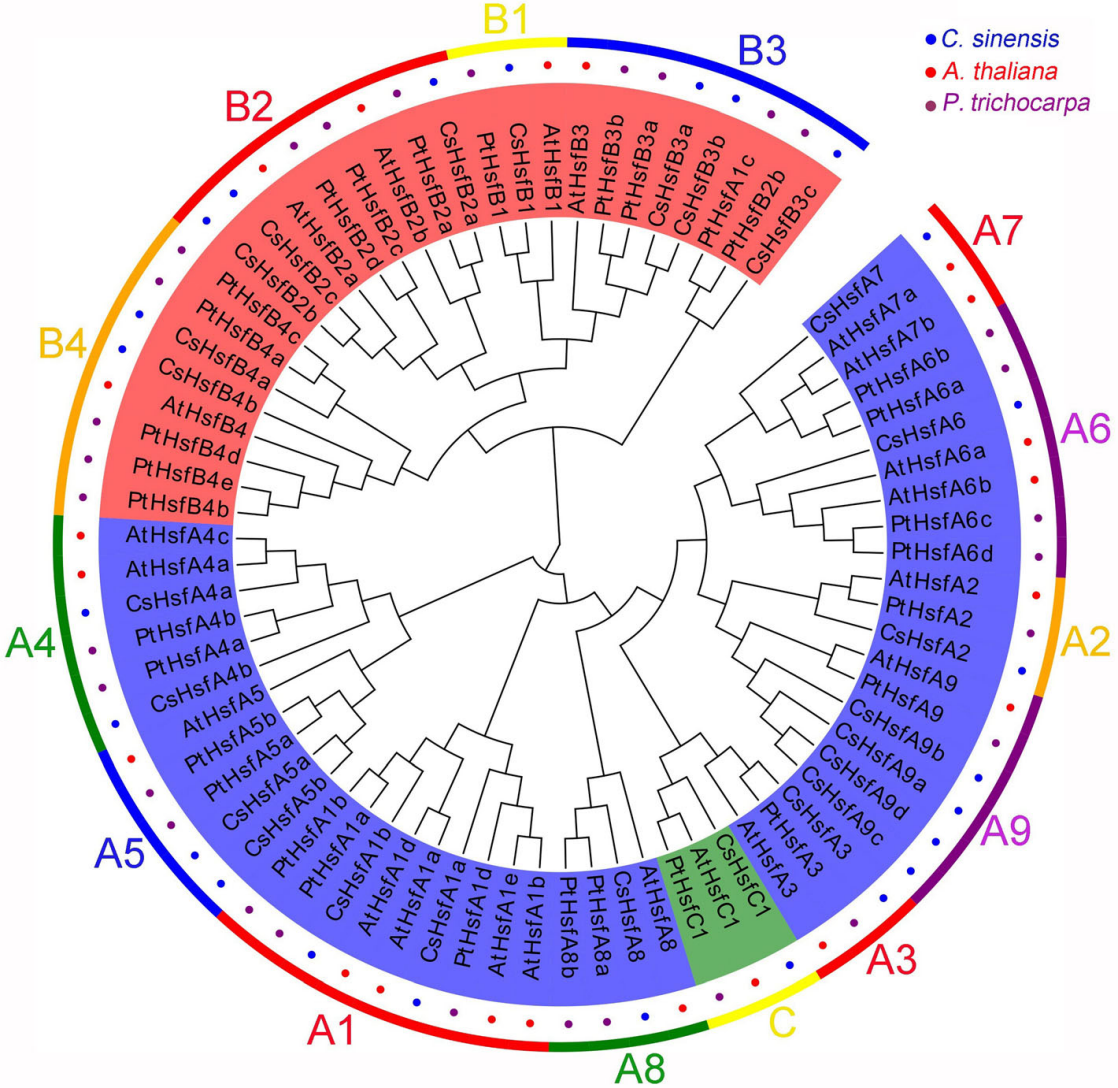
Phylogenetic tree of the Hsf TFs from *C. sinensis*, *A. thaliana*, and *P. trichocarpa*. Both multiple sequences alignment and phylogenetic tree were performed by MEGA (version 7.0) with the maximum likelihood method (1000 replicates). Different colors represent different Hsf subfamilies

To evaluate the evolutionary relationships among the Hsf family, a phylogenetic tree was generated using the Hsf sequences of 25 proteins from *C. sinensis*, 21 from *A. thaliana*, and 30 from *P. trichocarpa* (Fig. 2). According to this tree, the CsHsfs could be divided into three main classes (i.e., A, B, and C). Class A included 15 members from nine subclasses (i.e., A1–A9), Class B was divided into subclasses B1, B2, B3, and B4, while class C contains only one member (i.e., CsHsfC1). Interestingly, two *P. trichocarpa* Hsfs (i.e., PtHsfA1c and PtHsfB2b) were not clustered with their corresponding subclasses, but were closer to the HsfA3 subclass. In addition, compared with *A. thaliana*, *P. trichocarpa* Hsfs were closer to *C. sinensis* Hsf proteins.

### Gene structures and *cis*-elements analyses

**Fig. 3:**
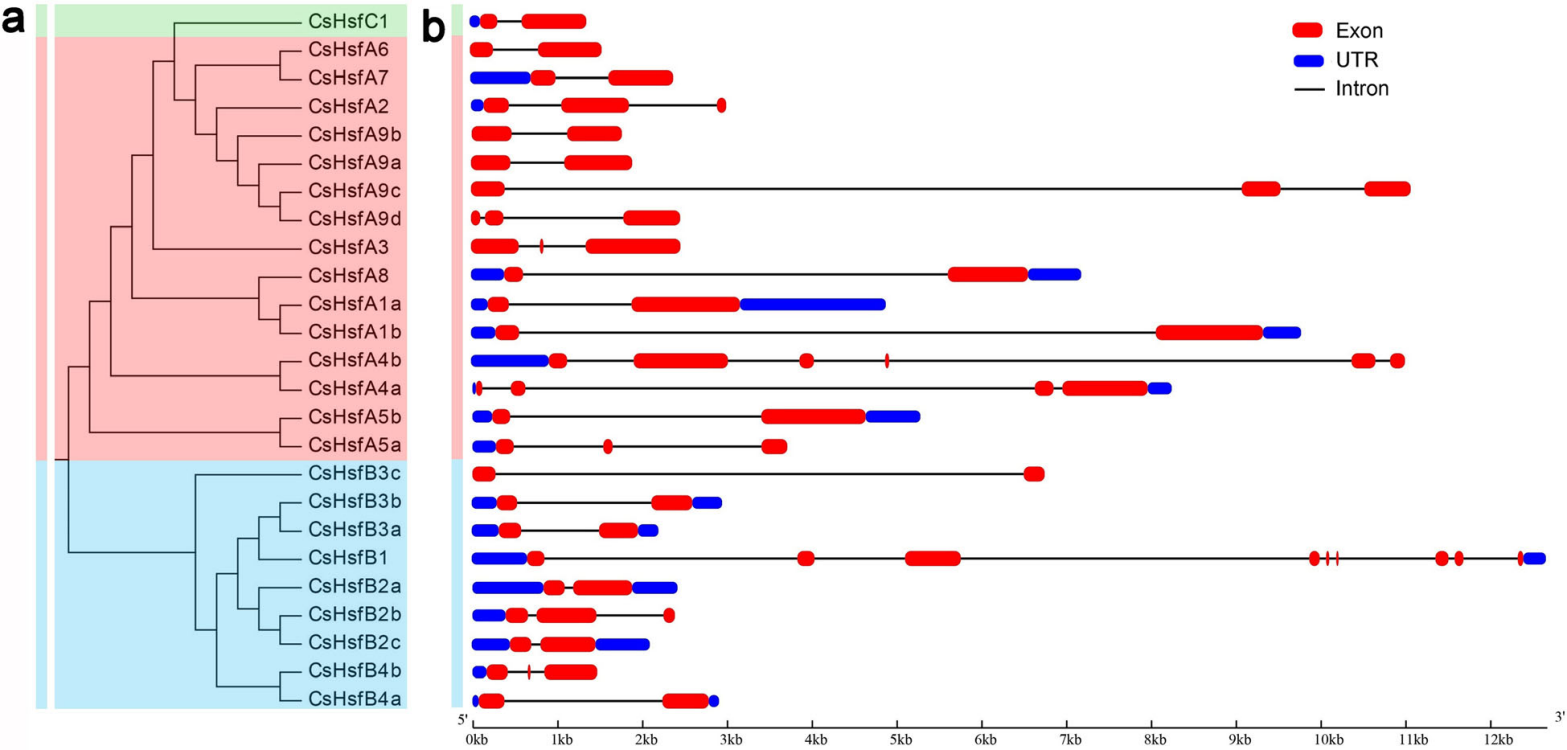
Phylogenetic tree and gene structure of the CsHsfs. **a** Phylogenetic tree was constructed by MEGA (version 7.0) with the maximum likelihood method (1000 replicates). Different colors represent different CsHsf subfamilies. **b** Exon/intron structures of *CsHsf* genes . Exons, UTRs, and introns are represented by the red round-cornered rectangles, blue round-cornered rectangles, and single lines, respectively. Lengths of exons, UTRs, and introns of each *CsHsf* gene are displayed proportionally

The structures of *CsHsf* genes were analyzed by comparing their cDNA sequences and genomic DNA sequences. Generally, most of the *CsHsf* genes contained one or two introns, while *CsHsfA4b* and *CsHsfB1* were comprised of five and eight introns, respectively (Fig. 3).

**Fig. 4:**
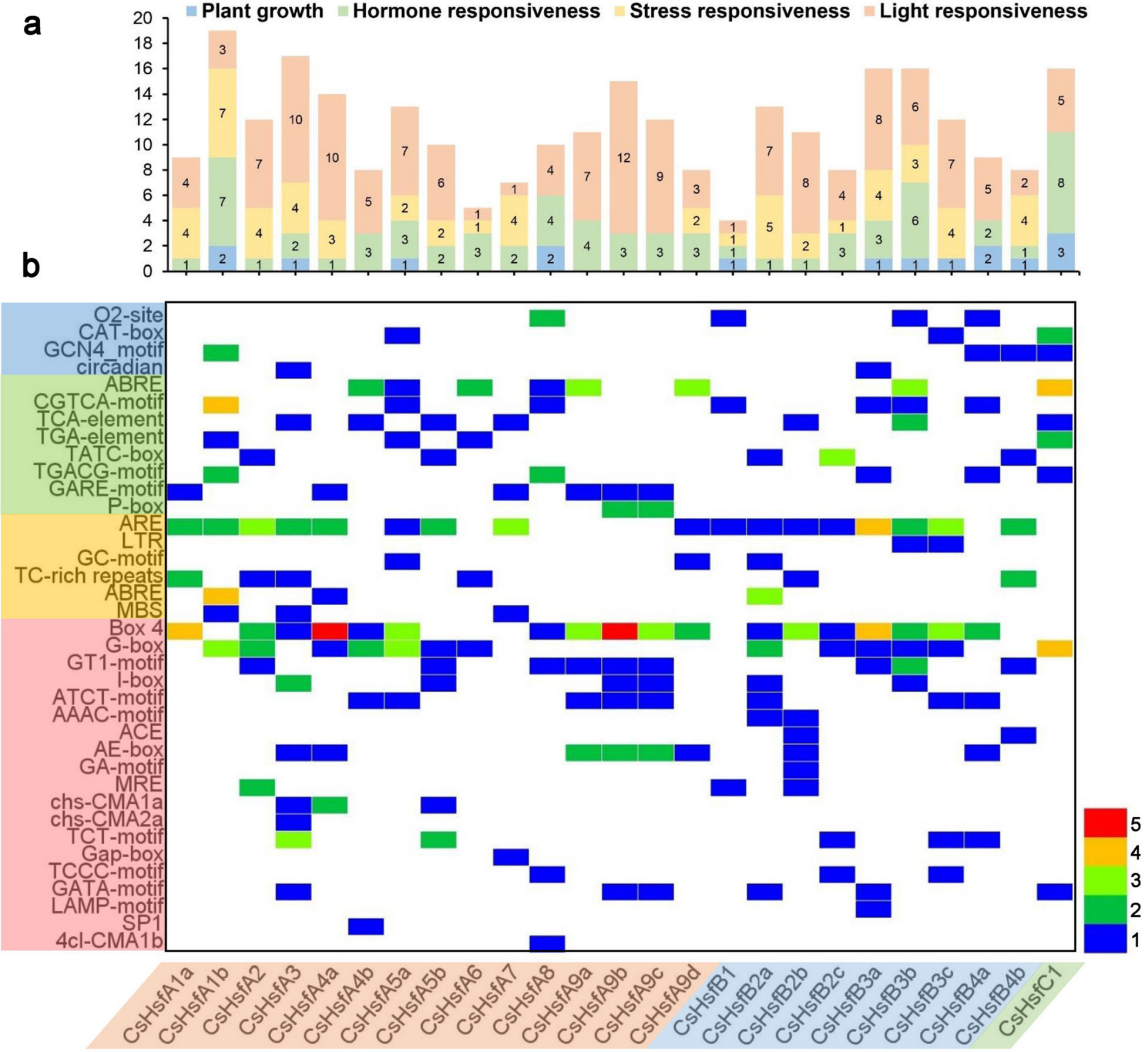
Analysis of the *cis*-elements in the promoter regions of the *CsHsf* genes. **a** The sum of the *cis*-elements in each *CsHsf* in four categories are represented by different colored histograms. **b** Heatmap of the number of *cis*-elements in each *CsHsf*. PlantCARE program (http://bioinformatics.psb.ugent.be/webtools/plantcare/html/) was used to perform the analysis. The different colors indicate the numbers of *cis*-elements, and the white represents no *cis*-element. Promoter sequences (2000 bp upstream of the translation start site) of *CsHsf* genes are listed in Additional file 2: Table S1

To further investigate the potential regulatory networks of the *CsHsf* family genes, the *cis*-elements in the 2 kb upstream sequences of the translation initiating site of 25 *CsHsf* genes were analyzed using PlantCARE [54] (Additional file 2: Table S1). A total of 37 types of *cis*-elements were discovered, including 4 plant growth regulation, 8 hormone responses, 6 stress responses, and 19 light responses elements (Fig. 4). Among the plant growth-related *cis*-acting elements, 4 *CsHsfs* had O2-site and CAT-box elements, 5 *CsHsfs* contained GCN4 motif, and 2 *CsHsfs* had circadian related elements. In the category of hormone responsiveness, a total of 19 abscisic acid responsive elements (ABRE), 17 MeJA-responsive elements (CGTCA-motif and TGACG-motif), 8 salicylic acid-responsive elements (TCA-element), 5 auxin-responsive elements (TGA-element), and 17 gibberellin-responsive elements (GARE-motif, TATC-box, and P-box) were detected in the promoter regions of 8, 8, 7, 4, and 11 *CsHsfs*, respectively. Moreover, many stress-responsive *cis*-elements were detected, including ARE (anaerobic induction element), LTR (low-temperature responsiveness), TC-rich repeats (defense and stress responsiveness), GC-motif (anoxic specific inducibility), and MBS (drought-inducibility). The light-responsive elements accounted for the largest part of all *cis*-elements, especially the Box 4 element, with a total of 48 distributed in 25 *CsHsf* promoter regions. In addition, G-box (23), AE-box (11), and GT1-motif (10) are involved in light responsiveness. Overall, these results indicated the involvement of *CsHsfs* in responses to hormone treatments, low temperature, and drought stresses in tea plants. Interestingly, no HSE element was detected in these *CsHsf* promoter regions. Hence, it remains unknown whether the expresssion of *CsHsf* genes was regulated by heat stress conditions.

### MiRNA target sites prediction

Tea plant degradome libraries (unpublished data) were used to predict target transcript candidates of *CsHsfs*. As shown in Additional file 3: Table S2, 7 *CsHsfs* are predicted to be targeted by 9 miRNAs. *CsHsfB3c* has 4 target sites, *CsHsfA4a* and *CsHsfA4b* have two target sites, while the other four CsHsfs (i.e., *CsHsfA1a*, *CsHsfA1b*, *CsHsfB1*, and *CsHsfA9d*) have only one target site. Hence, we inferred that a single miRNA can regulate multiple *CsHsfs* and a single *CsHsf* can be regulated by multiple miRNAs.

### Expression profiles of *CsHsf* genes in different tissues

**Fig. 5:**
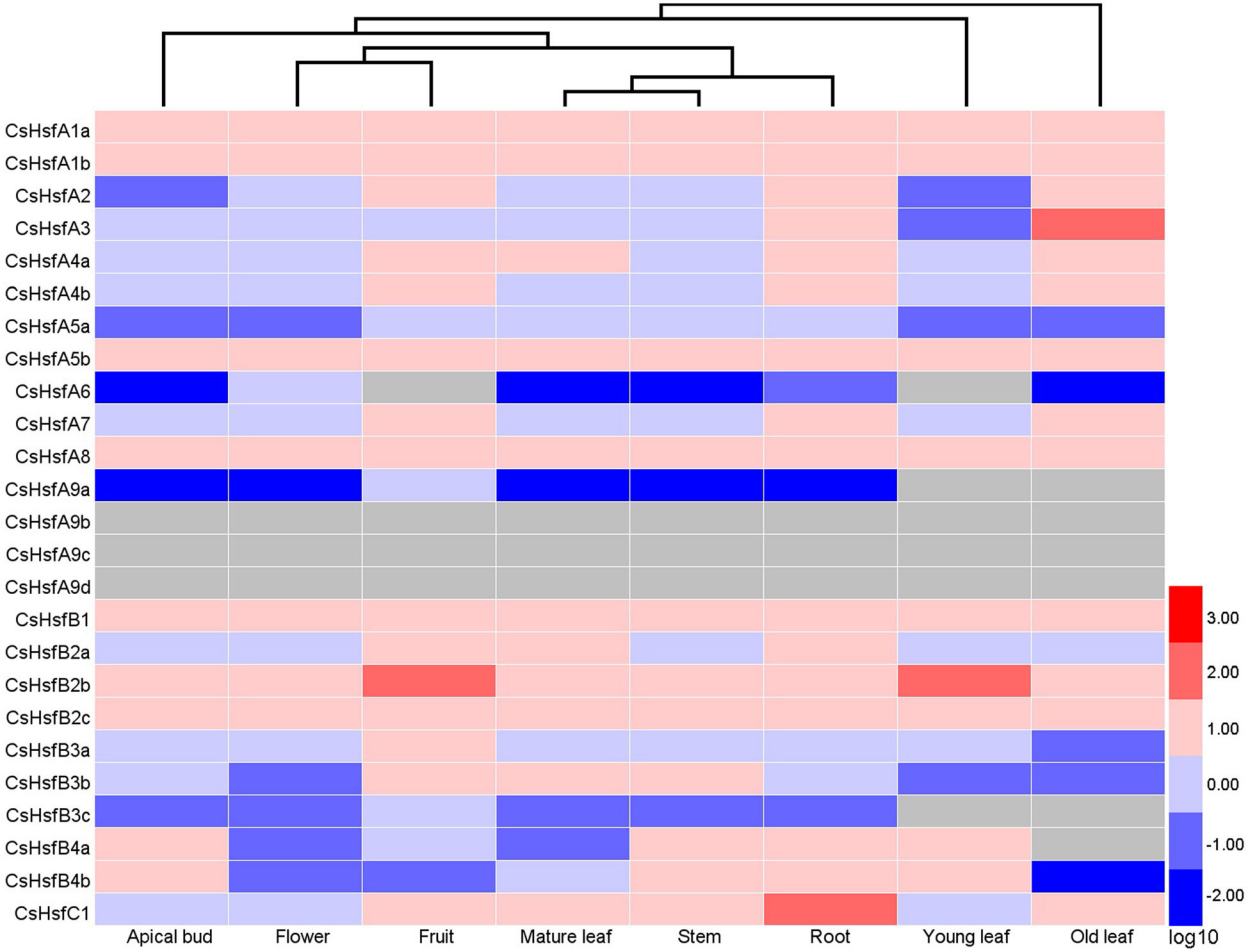
Expression profiles of *CsHsf* genes in tea plants (*C. sinensis* cv. ‘Shuchazao’) eight tissues based on the transcriptome data [47]. HemI software [56] was used to generate the heatmap. The color bar represents the normalized transcript per million (TPM) values (log10-transformed fold-changes). Red and blue colors indicate up- and down-regulated genes and the gray represents no expression. Detailed TPM values are listed in Additional file 4: Table S3

To examine the expression patterns of *CsHsf* genes among eight tissues, a heat map was drawn based on the transcriptome data downloaded from tea plant genome database [55]. The expression pattern of each *CsHsf* gene was significantly different in eight tissues (Fig. 5; Additional file 4: Table S3). *CsHsfA3* was highly expressed in old leaf (values > 2), *CsHsfB2b* was highly expressed in fruit and young leaf, and *CsHsfC1* was highly expressed in root. In comparison, *CsHsfA6*, *CsHsfB3c*, and *CsHsfB4a* were only expressed in some specific tissues. However, the expression of *CsHsfA9b*, *CsHsfA9c*, *CsHsfA9d* was undetectable in all tissues.

### Expression profiles of *CsHsf* genes in responses to drought and salt treatments

**Fig. 6:**
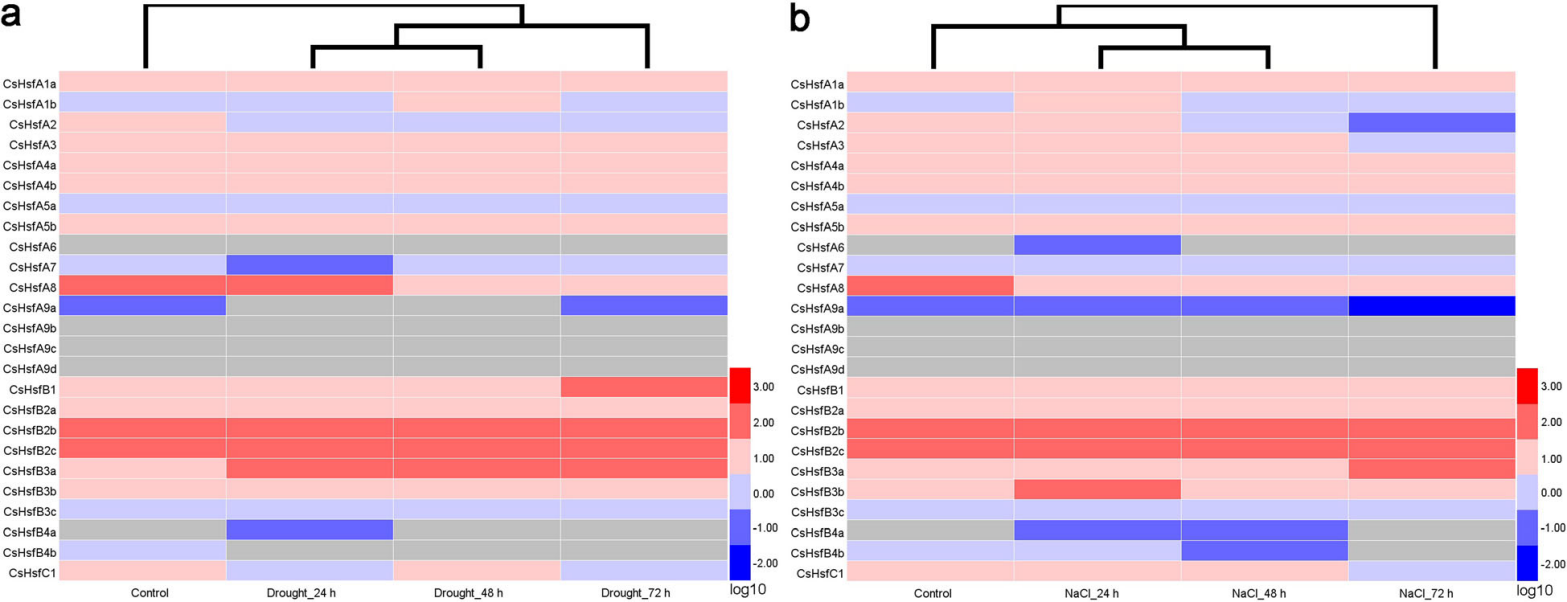
Expression profiles of *CsHsf* genes in tea plants (*C. sinensis* cv. ‘Tieguanyin’) exposed to (**a)** drought (25% polyethylene glycol 6000) and (**b)** salt (200 mM NaCl) treatments. HemI software [56] was used to generate the heatmap. The heatmap of *CsHsf* expression based on the transcriptome data [47]. The color bar represents the normalized transcript per million (TPM) values (log10-transformed fold-changes). Red and blue colors indicate up- and down-regulated genes and the gray represents no expression. Detailed TPM values are listed in Additional file 5: Table S4

To examine the roles of *CsHsf* genes in responses to drought and salt stresses, the transcriptome data of these genes were analyzed. Genes with the changes in transcription level greater than 1.5-fold were considered to be significantly regulated. After exposure to drought and salt stresses, most *CsHsfs* were up-regulated, whereas *CsHsfA1a* and *CsHsfB4a* were down-regulated (Fig. 6; Additional file 5: Table S4). *CsHsfB3a* was significantly up-regulated by drought stress (Fig. 6a), and *CsHsfA3*, *CsHsfA7*, *CsHsfB3a*, *CsHsfB3b*, *CsHsfB3c*, and *CsHsfB4b* were significantly up-regulated by salt stress (Fig. 6b). Interestingly, the expression level of *CsHsfA5a*, *CsHsfA7*, *CsHsfA9a*, *CsHsfB2c*, and *CsHsfB4b* showed the opposite trends under two abiotic stresses, indicating that they may have different roles in responses to drought and salt stresses in tea plants.

### Expression profiles of *CsHsf* genes in responses to heat and exogenous ABA treatments

**Fig. 7:**
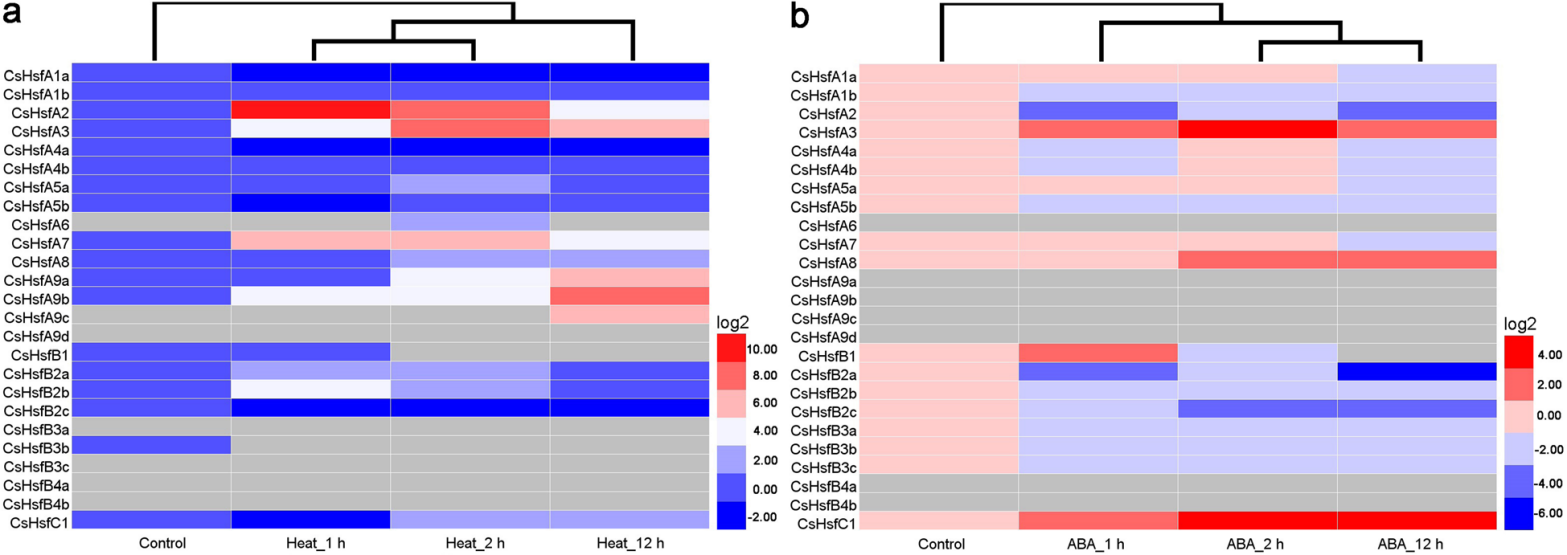
Expression profiles of *CsHsf* genes in tea plants (*C. sinensis* cv. ‘Echa 10’) exposed to (**a)** heat (38 °C) and (**b)** ABA (50 μM) treatments. HemI software [56] was used to generate the heatmap. The heatmap of *CsHsf* expression based on the qRT-PCR data of three biological and technical replicates. The color bar represents the normalized expression level (log2-transformed fold-changes). Red and blue colors indicate up- and down-regulated genes and the gray represents no expression. qRT-PCR data are listed in Additional file 6: Table S5

To further investigate the responses of *CsHsf* genes to heat and exogenous ABA treatments, we analyzed the expression profiles of *CsHsf* genes by qRT-PCR. When exposed to heat stress conditions, most *CsHsfs* were significantly up-regulated, especially *CsHsfA2* and *CsHsfA9b* (Fig. 7a; Additional file 6: Table S5). In contrast, *CsHsfA1a* and *CsHsfB3b* were down-regulated; however, the transcript abundance of *CsHsfA9d*, *CsHsfB3a*, *CsHsfB3c*, *CsHsfB4a*, and *CsHsfB4b* was too low to be detected. Following the exogenous ABA treatment, the transcript levels of *CsHsfA3*, *CsHsfA7*, *CsHsfA8*, *CsHsfB1*, and *CsHsfC1* increased significantly, while the transcripts of the other 9 *CsHsfs* (i.e., *CsHsfA1b*, *CsHsfA2*, *CsHsfA5b*, *CsHsfB2a*, *CsHsfB2b*, *CsHsfB2c*, *CsHsfB3a*, *CsHsfB3b*, and *CsHsfB3c*) were down-regulated (Fig. 7b; Additional file 6: Table S5). In addition, the transcript of *CsHsfA6*, *CsHsfA9a*, *CsHsfA9b*, *CsHsfA9c*, *CsHsfA9d*, *CsHsfB4a*, and *CsHsfB4b* was undetectable.

### Expression profiles of *CsHsf* genes with exogenous calcium treatments

**Fig. 8:**
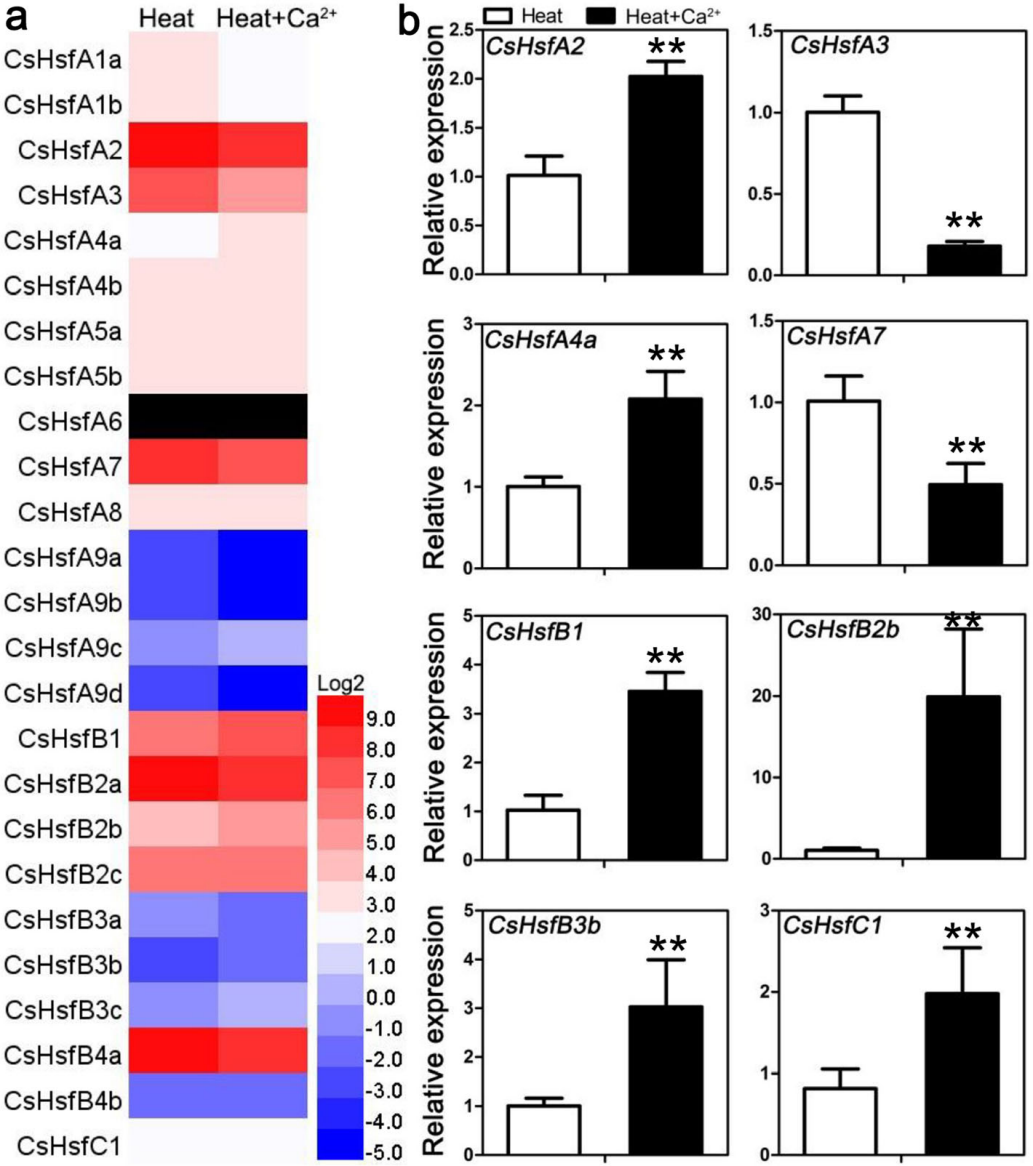
Expression profiles of *CsHsf* genes under heat stress conditions (38 °C for 4 h) in tea plants (*C. sinensis* cv. ‘Longjing-changyecha’) leaves pre-treated with exogenous calcium (20 mM). **a** Fragments per kilobase of transcript per million fragments mapped (FPKM) values [37] and HemI [56] were used to create the heat map. The color bar represents the normalized FPKM values (log2-transformed), and the black represents no expression detectable. Detailed FPKM values are listed in Additional file 7: Table S6. **b** Verification of the RNA-seq results of eight *CsHsf* genes by qRT-PCR analysis. Three biological replicates and three technical replicates were performed for the experiment. The qRT-PCR data was analyzed by ANOVA followed by Fisher’s LSD multiple comparison tests and ** represents significant differences at *P* < 0.01

To explore whether calcium ions (Ca^2+^) are involved in *Hsf*-mediating response to heat stress, we analyzed expression profiles of *CsHsf* genes in tea plants foliar-sprayed with exogenous Ca^2+^. According to RNA sequencing data, the fold changes of *CsHsfs* greater than 1.3-fold were considered to be significantly regulated. When exposure to exogenous Ca^2+^, the transcript abundance of *CsHsfA4a* was significantly up-regulated; and the transcription levels of the other four *CsHsfs* (i.e., *CsHsfA4b*, *CsHsfB1*, *CsHsfB2b*, *CsHsfB2c*) were slightly increased (Fig. 8a; Additional file 7: Table S6). Interestingly, the expression of the remaining *CsHsfs* was down-regulated. These results suggested that Ca^2+^ appeared to be involved in *Hsf*-mediating response to heat stress in tea plants. To further confirm the reliability of the RNA-seq results, four up-regulated and four down-regulated *CsHsf* genes were tested by qRT-PCR using the same tea plant variety (Fig. 8b; Additional file 8: Table S7). The results showed that the expression of six selected *CsHsfs* were well correlated with RNA-seq data, while the expression of *CsHsfA2* and *CsHsfC1* showed the opposite trends with RNA-seq results.

### CsHsfA2 localizes to the nucleus in onion epidermal cells

**Fig. 9:**
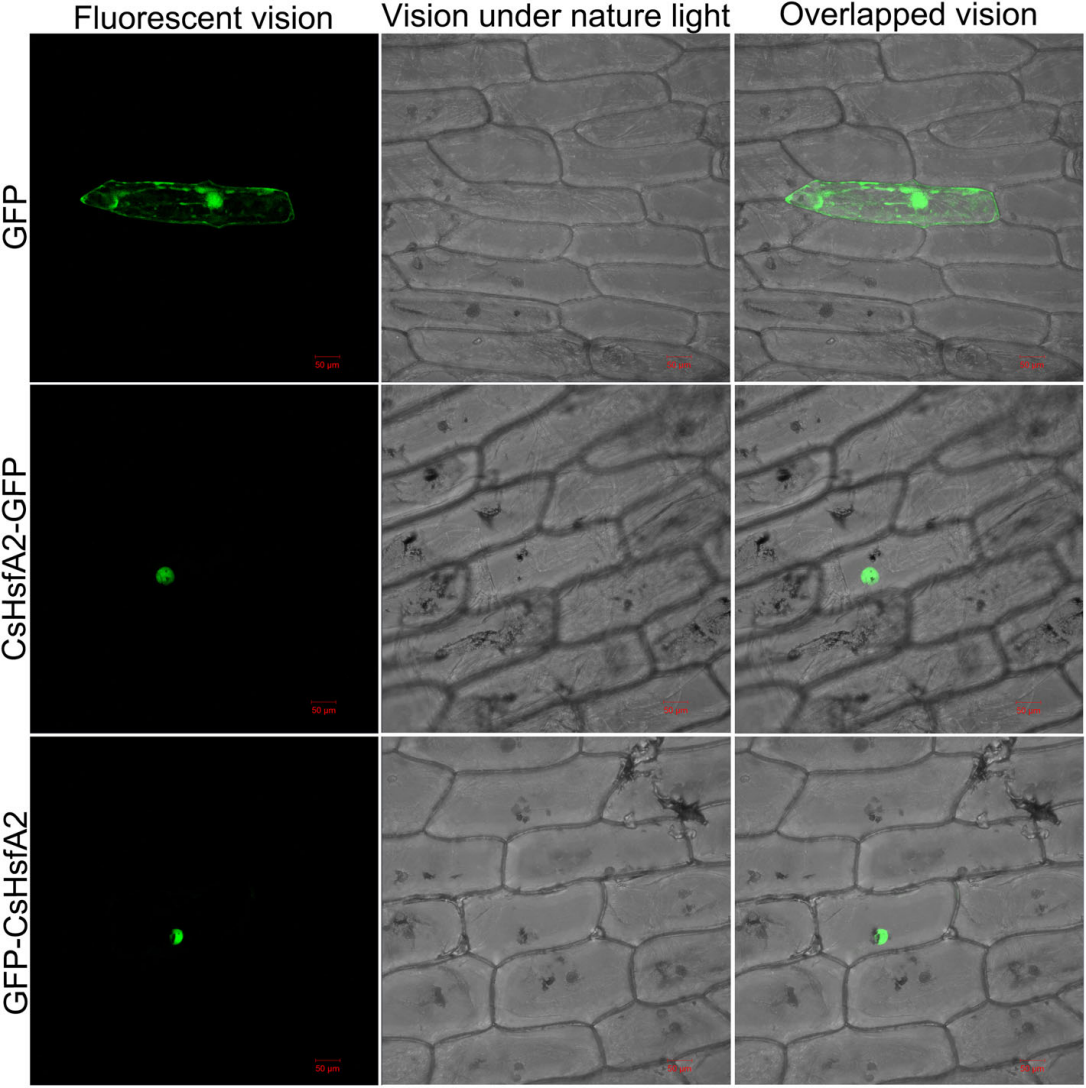
Subcellular localization of CsHsfA2 in onion epidermal cells. GFP indicates the vector control. CsHsfA2-GFP and GFP-CsHsfA2 indicate CsHsfA2 was fused with GFP at the C-terminus and N-terminus, respectively. The GFP signal was detected using a Zeiss LSM700 confocal laser-scanning microscope (Carl Zeiss Inc., USA) at 488 nm. Bars = 50 μm

*CsHsfA2* up-regulated strongly by heat stress was selected for subcellular localization analysis. As shown in Fig. 9, the control GFP signal was uniformly distributed throughout the cytosol and nucleus in onion epidermal cells, whereas the diffuse CsHsfA2-GFP and GFP-CsHsfA2 signals were only detected in the nucleus. Thus, CsHsfA2 is a nuclear protein, possibly serving as a transcription factor.

### Heterologous expression of *CsHsfA2* confers thermotolerance in transgenic yeast

**Fig. 10:**
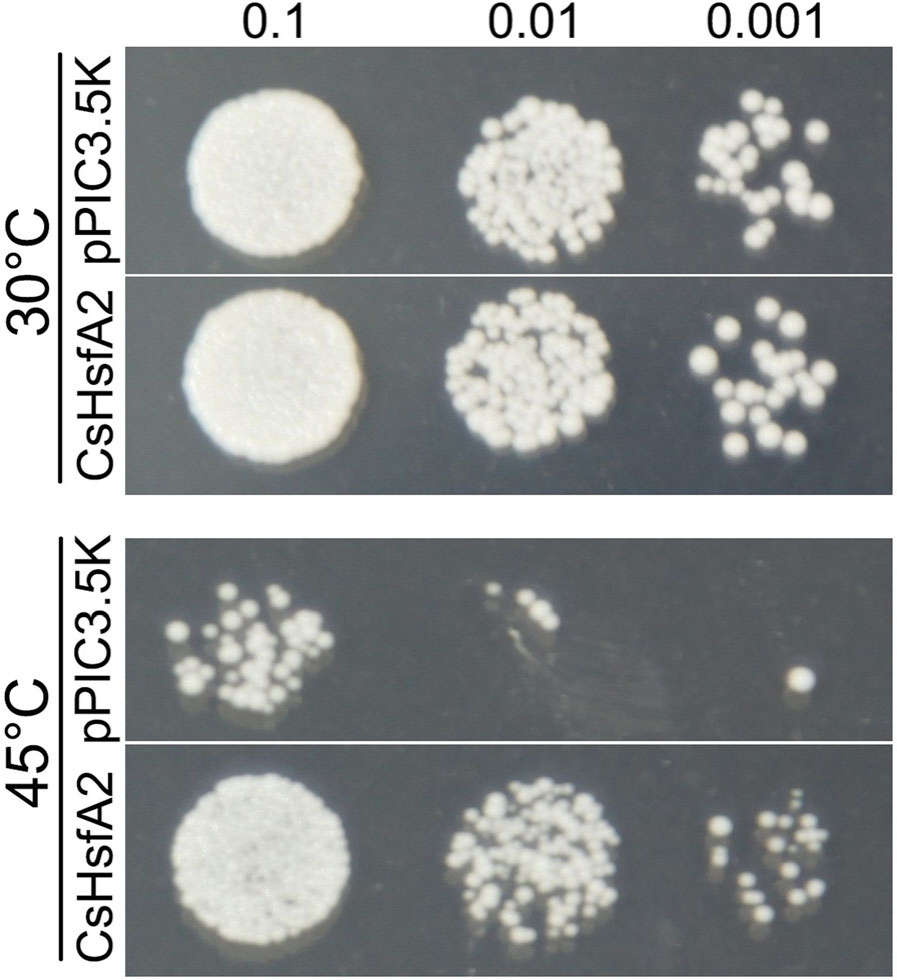
Heterologous expression of *CsHsfA2* confers thermotolerance in transgenic yeast. pPIC3.5 K and CsHsfA2 indicate the yeast cells transformed with the empty vector and recombinant plasmid, respectively. *P. pastoris* cells were exposed to heat stress (45 °C for 30 min), and then spotted onto YEPD medium plates (OD_600_ = 0.1, 0.01, and 0.001). The control samples were incubated under normal temperature conditions (30 °C)

Because of its dominant role in thermotolerance [9] and strongly up-regulated expression, we constructed a yeast expression vector and transformed it into yeast cell to evaluate the possible roles of *CsHsfA2* in response to heat stress. Under normal temperature conditions (30 °C), there were no obvious differences in yeast cells expressing *CsHsfA2* compared with the empty vector cells (i.e., pPIC3.5 K) (Fig. 10). However, when exposed to heat stress, the growth of yeast cells expressing *CsHsfA2* was better than the control cells. These results suggested that heterologous expression of *CsHsfA2* improved thermotolerance in transgenic yeast.

## Discussion

Tea plant, a perennial evergreen woody crop, has to cope with various abiotic stress during its lifecycle [57, 58]. Previous studies have showed that *Hsf* family genes play vital roles in responses to abiotic stress, especially high temperature stress [59]. Hence, it is necessary to investigate the Hsf family in tea plant. *C. sinensis Hsf* genes family were firstly identified according to the RNA-seq data by Liu et al. [46]. However, due to the limitations of transcriptome data with no reference genome, only 16 *CsHsfs* were identified. Here, we took advantage of the high quality tea plant reference genome [60] to identify and characterize the *CsHsfs* family bioinformatically.

The numbers of Hsf family are diverse in different plant species, and there are 25 *Hsfs* in pepper, 29 in Chinese white pear, 30 in sesame, 26 in soybean and 78 in bread wheat [12, 61–64], respectively. In this study, we identified 25 *CsHsfs* from tea plant genome and classified them into A, B, and C subfamily on the basis of their structures and phylogenetic relationships among *C. sinensis*, *A. thaliana*, *P. trichocarpa*. The number of subclass *HsfA9* members in tea plant enlarged with four members compared with only one member in *A. thaliana* and tomato (Table 1), which suggested the possibility of gene replication events during evolutionary process. Likely, the subclass *HsfA9* also had four members in pepper [64]. However, the expansion reasons of the *CsHsfA9* genes remain to be elucidated by further investigations. The theoretical isoelectric point of CsHsfB3c was 10.01 (Table 1), implying that it was a basic protein, and the other CsHsfs were acidic proteins, which indicated that they might play roles in different microenvironments [65]. The GRAVY results were all negative (Table 1), indicating that they were all hydrophilic proteins, which was consistent with the results in potato [66], carnation [67], and Chinese cabbage [68].

The highly conserved DBD domain consists of about 100 amino acid residues among various plant species [10]. However, it is noteworthy that the DBD of CsHsfA5a, CsHsfA9c, CsHsfA9d, and CsHsfB4b was shorter than the other CsHsfs (Fig. 1; Table 2), which may be attributed to the incomplete assembly of tea plant genome. AHA domain, which is specific to HsfA subgroup, is indispensable to activate the transcription of heat shock proteins (HSPs), but was not detected in CsHsfA5a and CsHsfA5b, suggesting that they might play their roles by binding to other HsfAs to form hetero oligomers [69]. Phylogenetic analysis showed that the CsHsfs could be divided into three main classes corresponding with those in *A. thaliana* and *P. trichocarpa* (Fig. 2), which was consistent with those reported previously [9, 61, 70]. The length of intron exhibited certain degrees of variation (Fig. 3), which was similar to that in other plants like potato [66] and carnation [67]. Additionally, some homologous genes had differences in intron numbers, intron length, and intron position, implying that their functions may be differentiated.

Promoter analysis indicated that the quantity and variety of *cis*-elements in each *CsHsf* gene were obviously different (Fig. 4), presumably suggesting that the transcription of *CsHsfs* may be differentially regulated by the combination of response elements. Moreover, no HSE element was detected in these *CsHsf* promoter regions, which implied that the expression of these heat-related *CsHsf* genes might not be directly induced by heat stress [66, 67]. The exact mechanism of gene expression needs further research. Further analysis of tea plant degradome data showed that 7 *CsHsfs* were predicted to be targeted by 9 miRNAs (Additional file 3: Table S2), implying that *CsHsfs* could be regulated at post-transcriptional level and miRNAs and their targets were not in one-to-one correspondence [71], but this hypothesis needs to be experimentally validated.

The exploration of gene expression patterns may help in understanding their biological functions [72]. In this study, the expression profiles of each *CsHsf* gene in eight tissues or exposure to different stresses (i.e., drought, salt, heat, and exogenous ABA) were investigated. Several *CsHsfs* showed tissue-specific expression patterns, such as *CsHsfA3* in old leaf, *CsHsfB2b* in fruit and young leaf, and *CsHsfC1* in root (Fig. 5), suggesting that these *CsHsfs* might be involved in the development of various organs and tissues. However, the expression of *CsHsfA9b*, *CsHsfA9c*, *CsHsfA9d* was undetectable in all tissues, including apical bud and young leaf, which was confirmed by qRT-PCR alalysis in control samples (one bud and two leaves), suggesting that the three members may have functional redundancy in tea plant. Previous studies have showed that the expression of *Hsfs* can be regulated by various abiotic stress, especially heat stress [22, 33, 73]. *AtHsfA2*, a key heat-inducible gene, could also be induced by salt and osmotic stresses in *A. thaliana* [74]. Likely, *LlHsfA2* expression could be induced by heat shock, but not by salt treatment in lily (*Lilium longiflorum*) [23]. Moreover, *CmHsfA4* was highly induced by salt stress in chrysanthemum [30]. In this study, the transcription of *CsHsfA2* was highly up-regulated by heat, salt, and drought stresses (Figs. 6, 7), but was down-regulated by exogenous ABA, suggesting its different roles in responses to various stresses. *AtHsfA3* was involved in heat and oxidative stresses responses [26, 75], while *CsHsfA3* could be induced by heat, salt, drought, and exogenous ABA. *CsHsfA7* was found to be up-regulated by heat shock stress, which was consistent with our previous findings [45]. Overexpression of *OsHsfA7* enhanced salt and drought tolerance in transgenic rice [76], while *AtHsfA7b* positively regulated salt stress tolerance in *A. thaliana* [31]. Hence, the specific functions of *CsHsfA7* in response to heat stress remain to be elucidated. TaHsfC2a-B played important roles in developing wheat grains via an ABA-dependent pathway in response to heat stress [33]. In addition to HS, *CsHsf* genes were also regulated by ABA (Fig. 7b). *CsHsfA3*, *CsHsfA8*, *CsHsfB1*, and *CsHsfC1* were up-regulated by exogenous ABA, while *CsHsfA2*, *CsHsfB2a*, and *CsHsfB2c* were down-regulated, suggesting that these *CsHsfs* played different roles in ABA-mediated regulatory pathway.

Ca^2+^ is a ubiquitous secondary messenger, and plays vital roles in response to a variety of environmental stresses [77, 78]. Here, exogenous Ca^2+^ pre-treatment induced the expression of *CsHsfA4a* under heat shock stress (Fig. 8), implying its potential roles in response to heat stress by Ca^2+^ signal pathway, but testing this hypothesis requires further research. Overexpression of *AtHsfA4A* resulted in tolerance to salt stress in *A. thaliana* [79]. Moreover, ectopic overexpression of *BnHSFA4a* in enhanced desiccation tolerance in *A. thaliana* seeds [80]. However, overexpression of sunflower *HaHsfA4a* alone did not confer thermotolerance in transgenic tobacco [81]. These results suggested the diverse roles of *HsfA4s* in responses to different abiotic stresses. Interestingly, the qRT-PCR results of *CsHsfA2* and *CsHsfC1* expression showed the opposite trends with RNA-seq results (Fig. 8), which was a normal phenomenon in other studies [46, 61, 64] and might be due to the different normalized method between FPKM and qRT-PCR.

Previous studies have demonstrated that the majority of Hsfs were localized to the nucleus [64]. In our study, CsHsfA5a and CsHsfA8 were predicted to be localized in the cytoplasm and chloroplast, respectively, while the other 23 CsHsfs were targeted to the nucleus (Table 1). To further confirm the subcellular localization prediction, CsHsfA2, CsHsfA5a, and CsHsfA8 were selected and transiently expressed in onion epidermal cells. Interestingly, CsHsfA2, CsHsfA5a, and CsHsfA8 fusion proteins were localized in the nucleus of onion epidermal cells (Fig. 9; Additional file 9: Figure S2). Hence, the subcellular localization prediction results could not reflect the true locations of target proteins, and needed to be verified by experiments. It is worth noting that *HsfA2* is a key heat-responsive gene in resistance to heat stress [9, 22, 82]. Since tea plant has no genetic transformation system available, we heterologously expressed *CsHsfA2* in eukaryotic model organism yeast to dissect the biological function of *CsHsfA2* in response to heat stress. Thermotolerance assays indicated that heterologous expression of *CsHsfA2* improved yeast resistance to high temperature (Fig. 10). To further elucidate the functions and regulation mechanisms of *CsHsfA2* in tea plant through virus-induced gene silencing, as underway in our lab, we would be able to explain the specific molecular mechanisms of *CsHsfA2* regulating tea plant heat response.

## Conclusions

In this study, we identified and comparatively analyzed 25 full-length tea plant *Hsfs* in both coding sequences and gene-expression profiles, as well as their expression patterns in responses to abiotic stress. It is worth noting that Ca^2+^ signal and ABA pathway seemed to be involved in the *CsHsf*-mediated heat response. Moreover, CsHsfA2 was located in the nucleus. Additionally, *CsHsfA2* conferred thermotolerance when heterologous expressed in transgenic yeast. All these results provide useful information for elaborating the *Hsf*-mediated stress-response system in tea plant as well as other plant species.

## Methods

### Identification and sequence analysis of *Hsf* genes from *C. sinensis*

The amino acid sequences of 21 *A. thaliana Hsf* genes were downloaded from National Center for Biotechnology Information (NCBI) database (https://www.ncbi.nlm.nih.gov/) as queries to search against the *C. sinensis* var. *sinensis* genome [60]. Furthermore, all obtained CsHsf proteins were analyzed to detect DBD domains and coiled-coil structures by the SMART [50] and CCD programs (https://www.ncbi.nlm.nih.gov/cdd/). Finally, to verify the accuracy of these sequences, BLASTN similarity searches against the published data of *C. sinensis* were performed with a threshold E-value of less than 1.0E− 90.

The physicochemical parameters of CsHsf proteins were calculated using the ExPASy program (http://web.expasy.org/compute_pi/) [47] with default parameters. WoLF PSORT (https://wolfpsort.hgc.jp/) [48] was used to predict subcellular localizations of the CsHsf proteins. The typical functional structure domains were analyzed using SMART [50], Pfam (http://pfam.xfam.org/search) [51], NLStradamus [52] and NetNES 1.1 server [53]. Multiple alignments of CsHsf DNA-binding domain (DBD) were analyzed using MultAlin [83]. The MEME (http://meme-suite.org/tools/meme) [49] and WebLogo (http://weblogo.berkeley.edu/logo.cgi) [84] programs were used to analyze and visualize the CsHsf conserved motifs with optimum motif width ≥ 6 bp and ≤ 200 bp and maximum number of motifs = 25. Phylogenetic trees were constructed using the neighbour-joining method in MEGA (version 7.0) software and bootstrap test replicate was set to 1000 [85]. The structures of *CsHsf* genes were analyzed using the online Gene Structure Display Server [86]. Furthermore, the *cis*-regulatory elements in the 2000 bp promoter regions of *CsHsf* genes were analyzed in the PlantCARE program [54], and visualized the number of *cis*-elements in each *CsHsf* on the HemI software [56]. In addition, psRNATarget online tool [87] was used to predict the miRNAs targeting the *CsHsf* genes according to the degradome libraries constructed in our lab (unpublished data).

### Expression profiles of *CsHsf* genes based on transcriptome data

To study the expression of *CsHsf* genes in eight tissues (i.e., apical bud, flower, old leaf, young leaf, fruit, mature leaf, stem, and root) and responses to drought and salt stresses, the *C. sinensis* expression data were downloaded from Tea Plant Information Archive (TPIA) [55]. The TPM (Transcript per million) values of each *CsHsf* gene were identified and log10 transformed.

The transcription of each *CsHsf* under heat stress in *C. sinensis* leaves treated with exogenous calcium was calculated using RNA sequencing data [37]. The expression profiles of each *CsHsf* were visualized using the HemI software [56].

### Plant materials and treatments

One-year-old cutting seedlings of tea plants [*Camellia sinensis* (L.) O. Ktze. ‘Echa No. 10’; an individual of ‘Enshi-taizicha’ group species] were cultivated in a growth chamber at Huazhong Agricultural University (Wuhan, China) with a photoperiod of 12 h light (24 ± 1 °C)/12 h dark (20 ± 1 °C) for 22 days before treatments. To simulate heat stress, the plantlets were placed in an illumination incubator (38 °C). For ABA treatment, the plantlets were treated with 50 μM ABA as described by Wang et al. [58]. Young shoots (one bud and two leaves) were harvested at 0, 1, 2, and 12 h after each treatment, immediately immersed in liquid nitrogen, and stored at − 70 °C prior to RNA extraction. Additionally, exogenous Ca^2+^ pre-treatment (20 mM CaCl_2_) was conducted as described previously [37], and the samples were collected after exposure to heat stress for 4 h. Three plants were pooled and taken as one biological replicate and three biological replicates were used.

### Quantitative RT-PCR analysis of *CsHsf* genes in *C. sinensis*

Total RNA was extracted using the Quick RNA Isolation Kit (Huayueyang, Beijing, China). Equal amounts of total RNA (1 μg) in all samples were treated with DNase I to eliminate genomic DNA contamination, and then used for cDNA synthesis using a *TransScript*® II All-in-One First-strand cDNA Synthesis SuperMix for qPCR (One-Step gDNA) Kit (TransGen, Beijing, China). The resulting cDNA was diluted 25-fold in distilled deionized water for qRT-PCR assay. The qRT-PCR assays were performed as described by Wang et al. [88] in a StepOne Plus™ Real-Time PCR System (Applied Biosystems, Foster City, CA, USA). Gene-specific primers (Additional file 10: Table S8) were designed according to the Minimum Information for Publication of Quantitative Real-Time PCR Experiments guidelines [89]. *C. sinensis β-actin* (Genbank accession number HQ420251) served as the internal reference gene for qRT-PCR normalization analysis, and the relative expression levels of *CsHsf* genes were calculated using the 2^–ΔΔCT^ method [90]. All experiments were conducted with three biological and three technical replicates.

### Subcellular localization of CsHsfA2, CsHsfA5a, and CsHsfA8

The coding regions of *CsHsfA2*, *CsHsfA5a*, and *CsHsfA8* without stop codon was firstly fused to the plant expression vector pCAMBIA2300-C-GFP and pCAMBIA2300-N-GFP using a Seamless Assembly and Cloning Kit (Aidlab, Beijing, China), respectively. Then, the onion epidermal cells were transformed and detected as described by Wang et al. [58].

### Thermotolerance analysis of transgenic yeast

The ORF of *CsHsfA2* was inserted into the pPIC3.5 K yeast expression vector (Invitrogen, Carlsbad, CA), and then the recombinant plasmid was transformed into *Pichia pastoris* SMD1168 competent cells (Invitrogen) using the freeze-thaw method. The thermotolerance assays were conducted as described by Jiang et al. [91] with minor modifications. Briefly, *P. pastoris* cells harboring the recombinant plasmid (OD_600_ = 1.0) or pPIC3.5 K were incubated in a water bath at 45 °C for 30 min, and then the yeast cells were spotted onto YEPD medium plates after 10-fold dilutions. The photograph was taken after 3 d cultivation under normal temperature conditions (30 °C).

## Supplementary information

**Additional file 1: Figure S1.** The distribution of conserved motifs and their corresponding sequence logos of CsHsf proteins. **a** 25 conserved motifs of CsHsf proteins were analyzed by MEME (http://meme-suite.org/tools/meme). **b** Sequence logos of the 25 conserved motifs.

**Additional file 2: Table S1.** Promoter sequences (2000 bp upstream of the translation start site) of CsHsf genes.

**Additional file 3: Table S2.** List of predicted miRNA target sites of *CsHsf* genes.

**Additional file 4: Table S3.** The transcript per million (TPM) values [47] of CsHsf genes in *C. sinensis* cv. ‘Shuchazao’ different tissues.

**Additional file 5: Table S4.** The transcript per million (TPM) values [47] of CsHsf genes exposed to drought (25% polyethylene glycol 6000) and salt (200 mM NaCl) treatments in *C. sinensis* cv. ‘Tieguanyin’.

**Additional file 6: Table S5.** Transcription levels of CsHsf genes exposed to heat and ABA treatments. Tea plant cultivar ‘Echa 10’ was used in the experiment and qRT-PCR data are presented as the mean ± SD of three biological and technical replicates.

**Additional file 7: Table S6.** The fragments per kilobase of transcript per million fragments mapped (FPKM) values [37] of CsHsf genes under heat stress conditions in tea plants (*C. sinensis* cv. ‘Longjing-changyecha’) leaves pre-treated with exogenous calcium.

**Additional file 8: Table S7.** Expression of eight CsHsf genes under heat stress conditions in tea plant (*C. sinensis* cv. ‘Longjing-changye’) leaves pre-treated with exogenous calcium. Bars indicate the standard deviation (SD) of the mean (*n* = 3). Each biological replicate contains three technical replicates.

**Additional file 9: Figure S2.** Subcellular localization of CsHsfA5a and CsHsfA8 in onion epidermal cells. CsHsfA5a-GFP and CsHsfA8-GFP indicate CsHsfA5a and CsHsfA8 fused with GFP at the C-terminus, and GFP-CsHsfA5a and GFP-CsHsfA8 indicate CsHsfA5a and CsHsfA8 fused with GFP at the N-terminus. The GFP signal was detected using a Zeiss LSM700 confocal laser-scanning microscope (Carl Zeiss Inc., USA) at 488 nm. Bars = 50 μm.

**Additional file 10: Table S8.** Primers used for qRT-PCR of CsHsf genes. These primers were specific for *C. sinensis* cv. ‘Echa 10’.

## Data Availability

All data generated or analyzed during this study are included in this article and its supplementary information files.

## Abbreviations

ABA: Abscisic acid
ABRE: Abscisic acid responsive elements
ARE: Anaerobic induction element
*A. thaliana*: *Arabidopsis thaliana*
Ca^2+^: Calcium ions
cDNA: Complementary DNA
*C. sinensis*: *Camellia sinensis*
DBD: DNA-binding domain
DNA: Deoxyribonucleic acid
DREB2A: Dehydration-responsive element-binding protein 2A
FPKM: Fragments per kilobase of transcript per million fragments mapped
gDNA: Genomic DNA
GFP: Green fluorescent protein
GRAVY: Grand average of hydropathicity
HS: Heat stress
HSE: HS elements
Hsfs: Heat stress factors
HSPs: Heat shock proteins
LTR: Low-temperature responsiveness
miRNA: MicroRNA
ORFs: Open reading frames
PEG: Polyethylene glycol
*P. pastoris*: *Pichia pastoris*
*P. trichocarpa*: *Populus trichocarpa*
qPCR: Quantitative polymerase chain reaction
qRT-PCR: Quantitative real-time PCR
TPIA: Tea Plant Information Archive
TPM: Transcript per million
YEPD: Yeast extract peptone dextrose medium

## References

[1] Xia EH, Tong W, Wu Q, Wei S, Zhao J, Zhang ZZ, Wei CL, Wan XC (2020). Tea plant genomics: achievements, challenges and perspectives. Hortic Res.

[2] Zhang X, He Y, He W, Su H, Wang Y, Hong G, Xu P (2019). Structural and functional insights into the LBD family involved in abiotic stress and flavonoid synthases in *Camellia sinensis*. Sci Rep.

[3] Shen W, Li H, Teng RM, Wang YX, Wang WL, Zhuang J (2019). Genomic and transcriptomic analyses of HD-zip family transcription factors and their responses to abiotic stress in tea plant (*Camellia sinensis*). Genomics.

[4] Wang YX, Liu ZW, Wu ZJ, Li H, Wang WL, Cui X, Zhuang J (2018). Genome-wide identification and expression analysis of GRAS family transcription factors in tea plant (*Camellia sinensis*). Sci Rep.

[5] Wang PJ, Chen D, Zheng YC, Jin S, Yang JF, Ye NX (2018). Identification and expression analyses of SBP-box genes reveal their involvement in abiotic stress and hormone response in tea plant (*Camellia sinensis*). Int J Mol Sci.

[6] Zhang Dayan, Han Zhaolan, Li Jinqiu, Qin Hao, Zhou Lin, Wang Yuhua, Zhu Xujun, Ma Yuanchun, Fang Wanping (2020). Genome-wide analysis of the SBP-box gene family transcription factors and their responses to abiotic stresses in tea (Camellia sinensis). Genomics.

[7] Wang PJ, Zheng YC, Guo YC, Chen XJ, Sun Y, Yang JF, Ye NX (2019). Identification, expression, and putative target gene analysis of nuclear factor-Y (NF-Y) transcription factors in tea plant (*Camellia sinensis*). Planta.

[8] Wang PJ, Chen XJ, Guo YC, Zheng YC, Yue C, Yang JF, Ye NX (2019). Identification of CBF transcription factors in tea plants and a survey of potential CBF target genes under low temperature. Int J Mol Sci.

[9] von Koskull-Doring P, Scharf KD, Nover L (2007). The diversity of plant heat stress transcription factors. Trends Plant Sci.

[10] Scharf KD, Berberich T, Ebersberger I, Nover L (2012). The plant heat stress transcription factor (Hsf) family: structure, function and evolution. Biochim Biophys Acta.

[11] Ohama N, Sato H, Shinozaki K, Yamaguchi-Shinozaki K (2017). Transcriptional regulatory network of plant heat stress response. Trends Plant Sci.

[12] Qiao X, Li M, Li LT, Yin H, Wu JY, Zhang SL (2015). Genome-wide identification and comparative analysis of the heat shock transcription factor family in Chinese white pear (*Pyrus bretschneideri*) and five other Rosaceae species. BMC Plant Biol.

[13] Ma J, Xu ZS, Wang F, Tan GF, Li MY, Xiong AS (2014). Genome-wide analysis of HSF family transcription factors and their responses to abiotic stresses in two Chinese cabbage varieties. Acta Physiol Plant.

[14] Lohani N, Golicz AA, Singh MB, Bhalla PL (2019). Genome-wide analysis of the *Hsf* gene family in *Brassica oleracea* and a comparative analysis of the *Hsf* gene family in *B. oleracea*, *B. rapa* and *B. napus*. Funct Integr Genomic.

[15] Xue GP, Sadat S, Drenth J, McIntyre CL (2014). The heat shock factor family from *Triticum aestivum* in response to heat and other major abiotic stresses and their role in regulation of heat shock protein genes. J Exp Bot.

[16] Zhang J, Li Y, Jia HX, Li JB, Huang J, Lu MZ, Hu JJ (2015). The heat shock factor gene family in *Salix suchowensis*: a genome-wide survey and expression profiling during development and abiotic stresses. Front Plant Sci.

[17] Zhu YX, Yan HW, Wang YY, Feng L, Chen Z, Xiang Y (2016). Genome duplication and evolution of heat shock transcription factor (HSF) gene family in four model angiosperms. J Plant Growth Regul.

[18] Zhou SJ, Zhang P, Jing ZG, Shi JL (2013). Genome-wide identification and analysis of heat shock transcription factor family in cucumber (*Cucumis sativus* L.). Plant Omics.

[19] Schramm F, Ganguli A, Kiehlmann E, Englich G, Walch D, von Koskull-Doring P (2006). The heat stress transcription factor HsfA2 serves as a regulatory amplifier of a subset of genes in the heat stress response in Arabidopsis. Plant Mol Biol.

[20] Hahn A, Bublak D, Schleiff E, Scharf KD (2011). Crosstalk between Hsp90 and Hsp70 chaperones and heat stress transcription factors in tomato. Plant Cell.

[21] Banti V, Mafessoni F, Loreti E, Alpi A, Perata P (2010). The heat-inducible transcription factor HsfA2 enhances anoxia tolerance in Arabidopsis. Plant Physiol.

[22] Li GL, Zhang HN, Shao HB, Wang GY, Zhang YY, Zhang YJ, Zhao LN, Guo XL, Sheteiwy MS (2019). ZmHsf05, a new heat shock transcription factor from *Zea mays* L. improves thermotolerance in *Arabidopsis thaliana* and rescues thermotolerance defects of the *athsfa2* mutant. Plant Sci.

[23] Xin HB, Zhang H, Chen L, Li XX, Lian QL, Yuan X, Hu XY, Cao L, He XL, Yi MF (2010). Cloning and characterization of *HsfA2* from lily (*Lilium longiflorum*). Plant Cell Rep.

[24] Yokotani N, Ichikawa T, Kondou Y, Matsui M, Hirochika H, Iwabuchi M, Oda K (2008). Expression of rice heat stress transcription factor OsHsfA2e enhances tolerance to environmental stresses in transgenic Arabidopsis. Planta.

[25] Zhu BG, Ye CJ, Lu H, Chen XJ, Chai G, Chen JN, Wang C (2006). Identification and characterization of a novel heat shock transcription factor gene, *GmHsfA1*, in soybeans (*Glycine max*). J Plant Res.

[26] Schramm F, Larkindale J, Kiehlmann E, Ganguli A, Englich G, Vierling E, von Koskull-Doring P (2008). A cascade of transcription factor DREB2A and heat stress transcription factor HsfA3 regulates the heat stress response of Arabidopsis. Plant J.

[27] Chen H, Hwang JE, Lim CJ, Kim DY, Lee SY, Lim CO (2010). Arabidopsis DREB2C functions as a transcriptional activator of HsfA3 during the heat stress response. Biochem Biophys Res Commun.

[28] Xiang JH, Ran J, Zou J, Zhou XY, Liu AL, Zhang XW, Peng Y, Tang N, Luo GY, Chen XB (2013). Heat shock factor OsHsfB2b negatively regulates drought and salt tolerance in rice. Plant Cell Rep.

[29] Reddy PS, Kishor PBK, Seiler C, Kuhlmann M, Eschen-Lippold L, Lee J, Reddy MK, Sreenivasulu N (2014). Unraveling regulation of the small heat shock proteins by the heat shock factor HvHsfB2c in barley: its implications in drought stress response and seed development. PLoS One.

[30] Li F, Zhang HR, Zhao HS, Gao TW, Song AP, Jiang JF, Chen FD, Chen SM (2018). Chrysanthemum *CmHSFA4* gene positively regulates salt stress tolerance in transgenic chrysanthemum. Plant Biotechnol J.

[31] Zang DD, Wang JX, Zhang X, Liu ZJ, Wang YC (2019). Arabidopsis heat shock transcription factor HSFA7b positively mediates salt stress tolerance by binding to an E-box-like motif to regulate gene expression. J Exp Bot.

[32] Schmidt R, Schippers JHM, Welker A, Mieulet D, Guiderdoni E, Mueller-Roeber B (2012). Transcription factor OsHsfC1b regulates salt tolerance and development in *Oryza sativa ssp. japonica*. AoB Plants.

[33] Hu XJ, Chen DD, Mclntyre CL, Dreccer MF, Zhang ZB, Drenth J, Kalaipandian S, Chang HP, Xue GP (2018). Heat shock factor C2a serves as a proactive mechanism for heat protection in developing grains in wheat *via* an ABA-mediated regulatory pathway. Plant Cell Environ.

[34] Prieto-Dapena P, Castano R, Almoguera C, Jordano J (2006). Improved resistance to controlled deterioration in transgenic seeds. Plant Physiol.

[35] Zhu XJ, Liao JR, Xia XL, Xiong F, Li Y, Shen JZ, Wen B, Ma YC, Wang YH, Fang WP (2019). Physiological and iTRAQ-based proteomic analyses reveal the function of exogenous γ-aminobutyric acid (GABA) in improving tea plant (*Camellia sinensis* L.) tolerance at cold temperature. BMC Plant Biol.

[36] Zhang QJ, Li W, Li K, Nan H, Shi C, Zhang Y, Dai ZY, Lin YL, Yang XL, Tong Y, et al. SMRT sequencing yields the chromosome-scale reference genome of tea tree, *Camellia sinensis var. sinensis*. bioRxiv. 2020; 10.1101/2020.01.02.892430.

[37] Wang ML, Zhang XY, Li QH, Chen X, Li XH (2019). Comparative transcriptome analysis to elucidate the enhanced thermotolerance of tea plants (*Camellia sinensis*) treated with exogenous calcium. Planta.

[38] Zhou L, Xu H, Mischke S, Meinhardt LW, Zhang DP, Zhu XJ, Li XH, Fang WP (2014). Exogenous abscisic acid significantly affects proteome in tea plant (*Camellia sinensis*) exposed to drought stress. Hortic Res.

[39] Liu SC, Jin JQ, Ma JQ, Yao MZ, Ma CL, Li CF, Ding ZT, Chen L (2016). Transcriptomic analysis of tea plant responding to drought stress and recovery. PLoS One.

[40] Wang H, Xu RK, Wang N, Li XH (2010). Soil acidification of alfisols as influenced by tea cultivation in eastern China. Pedosphere.

[41] Li SY, Li HX, Yang CL, Wang YD, Xue H, Niu YF (2016). Rates of soil acidification in tea plantations and possible causes. Agric Ecosyst Environ.

[42] Jin CW, Zheng SJ, He YF, Di Zhou G, Zhou ZX (2005). Lead contamination in tea garden soils and factors affecting its bioavailability. Chemosphere.

[43] Zhang MK, Fang LP (2007). Tea plantation-induced activation of soil heavy metals. Commun Soil Sci Plan.

[44] Wang Yong-Xin, Liu Zhi-Wei, Li Hui, Wang Wen-Li, Cui Xin, Zhuang Jing (2018). Understanding Response of Tea Plants to Heat Stress and the Mechanisms of Adaptation. Stress Physiology of Tea in the Face of Climate Change.

[45] Wang ML, Zou ZW, Li QH, Xin HH, Zhu XJ, Chen X, Li XH (2017). Heterologous expression of three *Camellia sinensis* small heat shock protein genes confers temperature stress tolerance in yeast and *Arabidopsis thaliana*. Plant Cell Rep.

[46] Liu ZW, Wu ZJ, Li XH, Huang Y, Li H, Wang YX, Zhuang J (2016). Identification, classification, and expression profiles of heat shock transcription factors in tea plant (*Camellia sinensis*) under temperature stress. Gene.

[47] Artimo P, Jonnalagedda M, Arnold K, Baratin D, Csardi G, de Castro E, Duvaud S, Flegel V, Fortier A, Gasteiger E (2012). ExPASy: SIB bioinformatics resource portal. Nucleic Acids Res.

[48] Horton P, Park KJ, Obayashi T, Obayashi T, Fujita N, Harada H, Adams-Collier CJ, Nakai K (2007). WoLF PSORT: protein localization predictor. Nucleic Acids Res.

[49] Bailey TL, Boden M, Buske FA, Frith M, Grant CE, Clementi L, Ren J, Li WW, Noble WS (2009). MEME SUITE: tools for motif discovery and searching. Nucleic Acids Res.

[50] Letunic I, Bork P (2018). 20 years of the SMART protein domain annotation resource. Nucleic Acids Res.

[51] El-Gebali S, Mistry J, Bateman A, Eddy SR, Luciani A, Potter SC, Qureshi M, Richardson LJ, Salazar GA (2019). The Pfam protein families database in 2019. Nucleic Acids Res.

[52] Ba ANN, Pogoutse A, Provart N, Moses AM (2009). NLStradamus: a simple hidden Markov model for nuclear localization signal prediction. BMC Bioinformatics.

[53] la Cour T, Kiemer L, Mølgaard A, Gupta R, Skriver K, Brunak S (2004). Analysis and prediction of leucine-rich nuclear export signals. Protein Eng Des Sel.

[54] Lescot M, Dehais P, Thijs G, Marchal K, Moreau Y, Van de Peer Y, Rouze P, Rombauts S (2002). PlantCARE, a database of plant *cis*-acting regulatory elements and a portal to tools for in silico analysis of promoter sequences. Nucleic Acids Res.

[55] Xia EH, Li FD, Tong W, Li PH, Wu Q, Zhao HJ, Ge RH, Li RP, Li YY, Zhang ZZ (2019). Tea plant information archive: a comprehensive genomics and bioinformatics platform for tea plant. Plant Biotechnol J.

[56] Deng WK, Wang YB, Liu ZX, Cheng H, Xue Y (2014). HemI: a toolkit for illustrating heatmaps. PLoS One.

[57] Duncan J, Saikia S, Gupta N, Biggs E (2016). Observing climate impacts on tea yield in Assam, India. Appl Geogr.

[58] Wang ML, Zou ZW, Li QH, Sun K, Chen X, Li XH (2017). The CsHSP17.2 molecular chaperone is essential for thermotolerance in *Camellia sinensis*. Sci Rep.

[59] Wang XM, Shi X, Chen SY, Ma C, Xu SB (2018). Evolutionary origin, gradual accumulation and functional divergence of heat shock factor gene family with plant evolution. Front Plant Sci.

[60] Wei CL, Yang H, Wang SB, Zhao J, Liu C, Gao LP, Xia EH, Lu Y, Tai YL, She GB (2018). Draft genome sequence of *Camellia sinensis* var. *sinensis* provides insights into the evolution of the tea genome and tea quality. Proc Natl Acad Sci U S A.

[61] Zhou M, Zheng SG, Liu R, Lu J, Lu L, Zhang CH, Liu ZH, Luo CP, Zhang L, Yant L (2019). Genome-wide identification, phylogenetic and expression analysis of the heat shock transcription factor family in bread wheat (*Triticum aestivum* L.). BMC Genomics.

[62] Chung E, Kim KM, Lee JH (2013). Genome-wide analysis and molecular characterization of heat shock transcription factor family in *Glycine max*. J Genet Genomics.

[63] Dossa K, Diouf D, Cisse N (2016). Genome-wide investigation of *Hsf* genes in sesame reveals their segmental duplication expansion and their active role in drought stress response. Front Plant Sci.

[64] Guo M, Lu JP, Zhai YF, Chai WG, Gong ZH, Lu MH (2015). Genome-wide analysis, expression profile of heat shock factor gene family (CaHsfs) and characterisation of CaHsfA2 in pepper (*Capsicum annuum* L.). BMC Plant Biol.

[65] Kiraga J, Mackiewicz P, Mackiewicz D, Kowalczuk M, Biecek P, Polak N, Smolarczyk K, Dudek MR, Cebrat S (2007). The relationships between the isoelectric point and: length of proteins, taxonomy and ecology of organisms. BMC Genomics.

[66] Tang R, Zhu W, Song X, Lin X, Cai J, Wang M, Yang Q (2016). Genome-wide identification and function analyses of heat shock transcription factors in potato. Front Plant Sci.

[67] Li W, Wan XL, Yu JY, Wang KL, Zhang J (2019). Genome-wide identification, classification, and expression analysis of the *Hsf* gene family in carnation (*Dianthus caryophyllus*). Int J Mol Sci.

[68] Song X, Liu G, Duan W, Liu T, Huang Z, Ren J, Li Y, Hou X (2014). Genome-wide identifcation, classifcation and expression analysis of the heat shock transcription factor family in Chinese cabbage. Mol Gen Genomics.

[69] Kotak S, Port M, Ganguli A, Bicker F, Von Koskull-Döring P (2004). Characterization of C-terminal domains of Arabidopsis heat stress transcription factors (Hsfs) and identification of a new signature combination of plant class a Hsfs with AHA and NES motifs essential for activator function and intracellular localization. Plant J.

[70] Chen SS, Jiang J, Han XJ, Zhang YX, Zhuo RY (2018). Identification, expression analysis of the Hsf family, and characterization of class A4 in *Sedum alfredii* hance under cadmium stress. Int J Mol Sci.

[71] Zhang Y, Zhu XJ, Chen X, Song CNA, Zou ZW, Wang YH, Wang ML, Fang WP, Li XH (2014). Identification and characterization of cold-responsive microRNAs in tea plant (*Camellia sinensis*) and their targets using high-throughput sequencing and degradome analysis. BMC Plant Biol.

[72] Maheswari U, Jabbari K, Petit JL, Porcel BM, Allen AE, Cadoret JP, De Martino A, Heijde M, Kaas R, La Roche J (2010). Digital expression profiling of novel diatom transcripts provides insight into their biological functions. Genome Biol.

[73] Bian XH, Li W, Niu CF, Wei W, Hu Y, Han JQ, Lu X, Tao JJ, Jin M, Qin H (2020). A class B heat shock factor selected for during soybean domestication contributes to salt tolerance by promoting flavonoid biosynthesis. New Phytol.

[74] Ogawa D, Yamaguchi K, Nishiuchi T (2007). High-level overexpression of the Arabidopsis *HsfA2* gene confers not only increased themotolerance but also salt/osmotic stress tolerance and enhanced callus growth. J Exp Bot.

[75] Song C, Chung WS, Lim CO (2016). Overexpression of heat shock factor gene *HsfA3* increases galactinol levels and oxidative stress tolerance in Arabidopsis. Mol Cells.

[76] Liu AL, Zou J, Liu CF, Zhou XY, Zhang XW, Luo GY, Chen XB (2013). Over-expression of *OsHsfA7* enhanced salt and drought tolerance in transgenic rice. BMB Rep.

[77] Tan W, Meng QW, Brestic M, Olsovska K, Yang XH (2011). Photosynthesis is improved by exogenous calcium in heat-stressed tobacco plants. J Plant Physiol.

[78] Lin KH, Huang SB, Wu CW, Chang YS (2019). Effects of salicylic acid and calcium chloride on heat tolerance of poinsettia. Hortscience.

[79] Perez-Salamo I, Papdi C, Rigo G, Zsigmond L, Vilela B, Lumbreras V, Nagy I, Horvath B, Domoki M, Darula Z (2014). The heat shock factor A4A confers salt tolerance and is regulated by oxidative stress and the mitogen-activated protein kinases MPK3 and MPK6. Plant Physiol.

[80] Lang SR, Liu XX, Xue H, Li X, Wang XF (2017). Functional characterization of BnHSFA4a as a heat shock transcription factor in controlling the re-establishment of desiccation tolerance in seeds. J Exp Bot.

[81] Personat JM, Tejedor-Cano J, Prieto-Dapena P, Almoguera C, Jordano J (2014). Co-overexpression of two heat shock factors results in enhanced seed longevity and in synergistic effects on seedling tolerance to severe dehydration and oxidative stress. BMC Plant Biol.

[82] Charng YY, Liu HC, Liu NY, Chi WT, Wang CN, Chang SH, Wang TT (2007). A heat-inducible transcription factor, HsfA2, is required for extension of acquired thermotolerance in Arabidopsis. Plant Physiol.

[83] Corpet F (1988). Multiple sequence alignment with hierarchical-clustering. Nucleic Acids Res.

[84] Crooks GE, Hon G, Chandonia JM, Brenner SE (2004). WebLogo: a sequence logo generator. Genome Res.

[85] Kumar S, Stecher G, Tamura K (2016). MEGA7: molecular evolutionary genetics analysis version 7.0 for bigger datasets. Mol Biol Evol.

[86] Hu B, Jin JP, Guo AY, Zhang H, Luo JC, Gao G (2015). GSDS 2.0: an upgraded gene feature visualization server. Bioinformatics.

[87] Dai XB, Zhuang ZH, Zhao PX (2018). psRNATarget: a plant small RNA target analysis server (2017 release). Nucleic Acids Res.

[88] Wang ML, Li QH, Xin HH, Chen X, Zhu XJ, Li XH (2017). Reliable reference genes for normalization of gene expression data in tea plants (*Camellia sinensis*) exposed to metal stresses. PLoS One.

[89] Bustin SA, Benes V, Garson JA, Hellemans J, Huggett J, Kubista M, Mueller R, Nolan T, Pfaffl MW, Shipley GL (2009). The MIQE guidelines: minimum information for publication of quantitative real-time PCR experiments. Clin Chem.

[90] Livak KJ, Schmittgen TD (2001). Analysis of relative gene expression data using real-time quantitative PCR and the 2^–ΔΔCT^ method. Methods.

[91] Jiang CH, Xu JY, Zhang H, Zhang X, Shi JL, Li M, Ming F (2009). A cytosolic class I small heat shock protein, RcHSP17.8, of *Rosa chinensis* confers resistance to a variety of stresses to *Escherichia coli*, yeast and *Arabidopsis thaliana*. Plant Cell Environ.

