# Supplementary material for: Genome-wide characterization of tea plant (Camellia sinensis) Hsf transcription factor family and role of CsHsfA2 in heat tolerance
Source: BMC Plant Biol. 2020 May 29;20:244. doi: 10.1186/s12870-020-02462-9 (PMC7260767; doi:10.1186/s12870-020-02462-9)
Supplement: Supplementary file 1 — Additional file 1: Figure S1. The distribution of conserved motifs and their corresponding sequence logos of CsHsf proteins. a 25 conserved motifs of CsHsf proteins were analyzed by MEME (http://meme-suite.org/tools/meme). b Sequence logos of the 25 conserved motifs. [file 12870_2020_2462_MOESM1_ESM.docx]

**
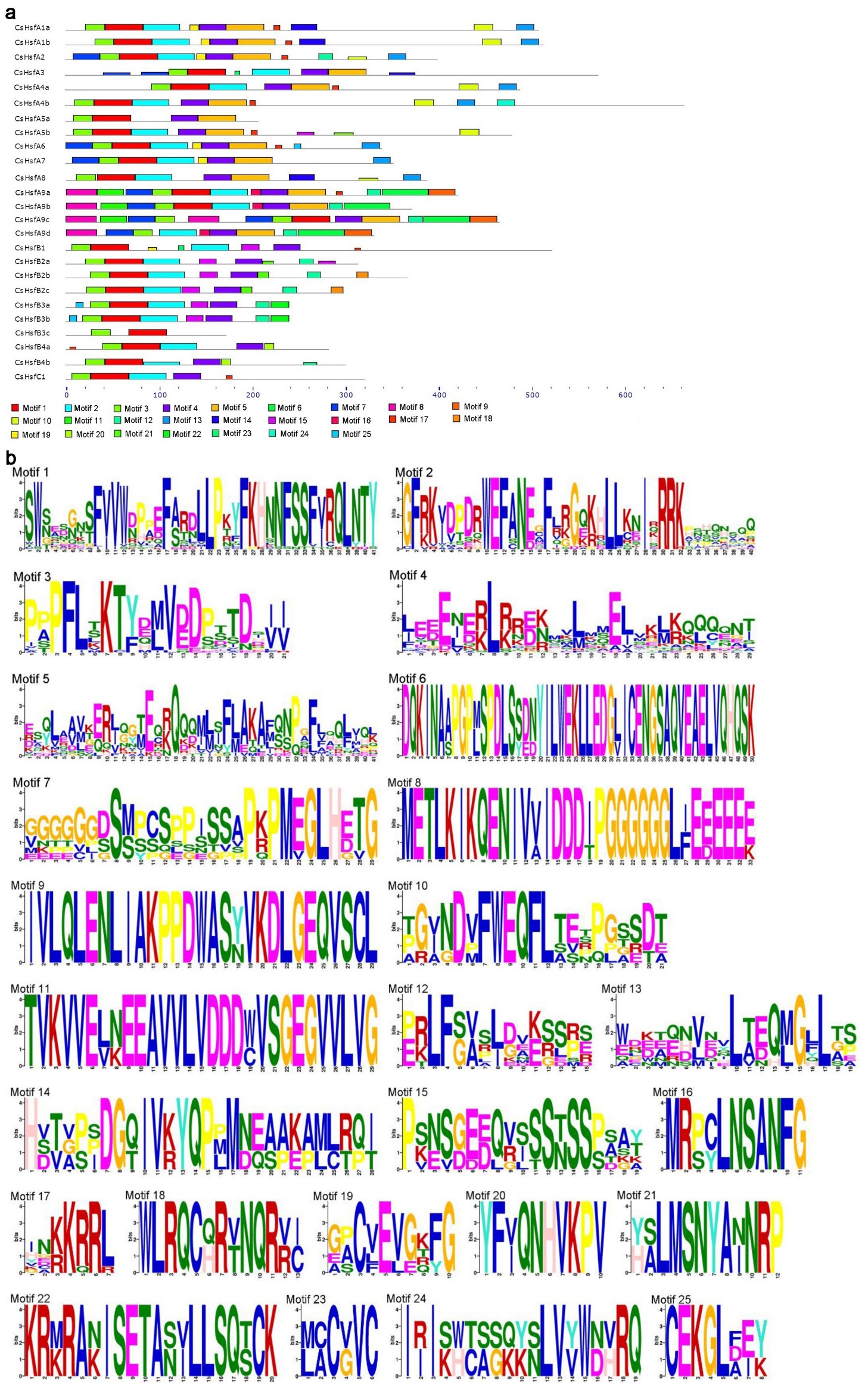
**

**Figure S1.** The distribution of conserved motifs and their corresponding sequence logos of CsHsf proteins. **a** 25 conserved motifs of CsHsf proteins were analyzed by MEME (http://meme-suite.org/tools/meme). **b** Sequence logos of the 25 conserved motifs
