# Supplementary material for: Genome-wide characterization of tea plant (Camellia sinensis) Hsf transcription factor family and role of CsHsfA2 in heat tolerance
Source: BMC Plant Biol. 2020 May 29;20:244. doi: 10.1186/s12870-020-02462-9 (PMC7260767; doi:10.1186/s12870-020-02462-9)
Supplement: Supplementary file 3 — Additional file 3: Table S2. List of predicted miRNA target sites of CsHsf genes. [file 12870_2020_2462_MOESM3_ESM.docx]

**Table S2.** List of predicted miRNA target sites of *CsHsf* genes

| **miRNA name** | **miRNA Sequence** | **Target gene** | **Gene ID** |
| --- | --- | --- | --- |
| csn-miR-10509 | GUGUUUGGUUGGAGGAUUUGAGG | *CsHsfB3c* | TEA010217 |
|  |  | *CsHsfA4b* | TEA014681 |
| csn-miR-2245 | AAGAUGUGUGAAACUAUGUGAAAU | *CsHsfB3c* | TEA010217 |
|  |  | *CsHsfA4b* | TEA014681 |
| csn-miR-7608 | UUGUGUUUUAUUUCUGUGCAAGUA | *CsHsfB3c* | TEA010217 |
| csn-miR-7902 | UUUGGAAAGGAAAAUGAAAAAGG | *CsHsfB3c* | TEA010217 |
| csn-miR-4646 | UUUGGAAAGGGAAAGGGAAAAGG | *CsHsfB3c* | TEA010217 |
|  |  | *CsHsfA1a* | TEA029045 |
|  |  | *CsHsfB1* | TEA013918 |
| csn-miR-7776 | GAUUAAUAGUGAGAGGAUUGUUAG | *CsHsfA9d* | TEA014078 |
| csn-miR-7649 | UUAGUAAACAAGCCGAGCUCGAGC | *CsHsfA4a* | TEA024058 |
| csn-miR-5993 | UCGACGAGUUCUACACAUUGAAUG | *CsHsfA4a* | TEA024058 |
| csn-miR-35w | CUCAAUAAACGAACUCGAUA | *CsHsfA1b* | TEA030860 |
