# Supplementary material for: Genome-wide characterization of tea plant (Camellia sinensis) Hsf transcription factor family and role of CsHsfA2 in heat tolerance
Source: BMC Plant Biol. 2020 May 29;20:244. doi: 10.1186/s12870-020-02462-9 (PMC7260767; doi:10.1186/s12870-020-02462-9)
Supplement: Supplementary file 9 — Additional file 9: Figure S2. Subcellular localization of CsHsfA5a and CsHsfA8 in onion epidermal cells. CsHsfA5a-GFP and CsHsfA8-GFP indicate CsHsfA5a and CsHsfA8 fused with GFP at the C-terminus, and GFP-CsHsfA5a and GFP-CsHsfA8 indicate CsHsfA5a and CsHsfA8 fused with GFP at the N-terminus. The GFP signal was detected using a Zeiss LSM700 confocal laser-scanning microscope (Carl Zeiss Inc., USA) at 488 nm. Bars = 50 μm. [file 12870_2020_2462_MOESM9_ESM.docx]

**
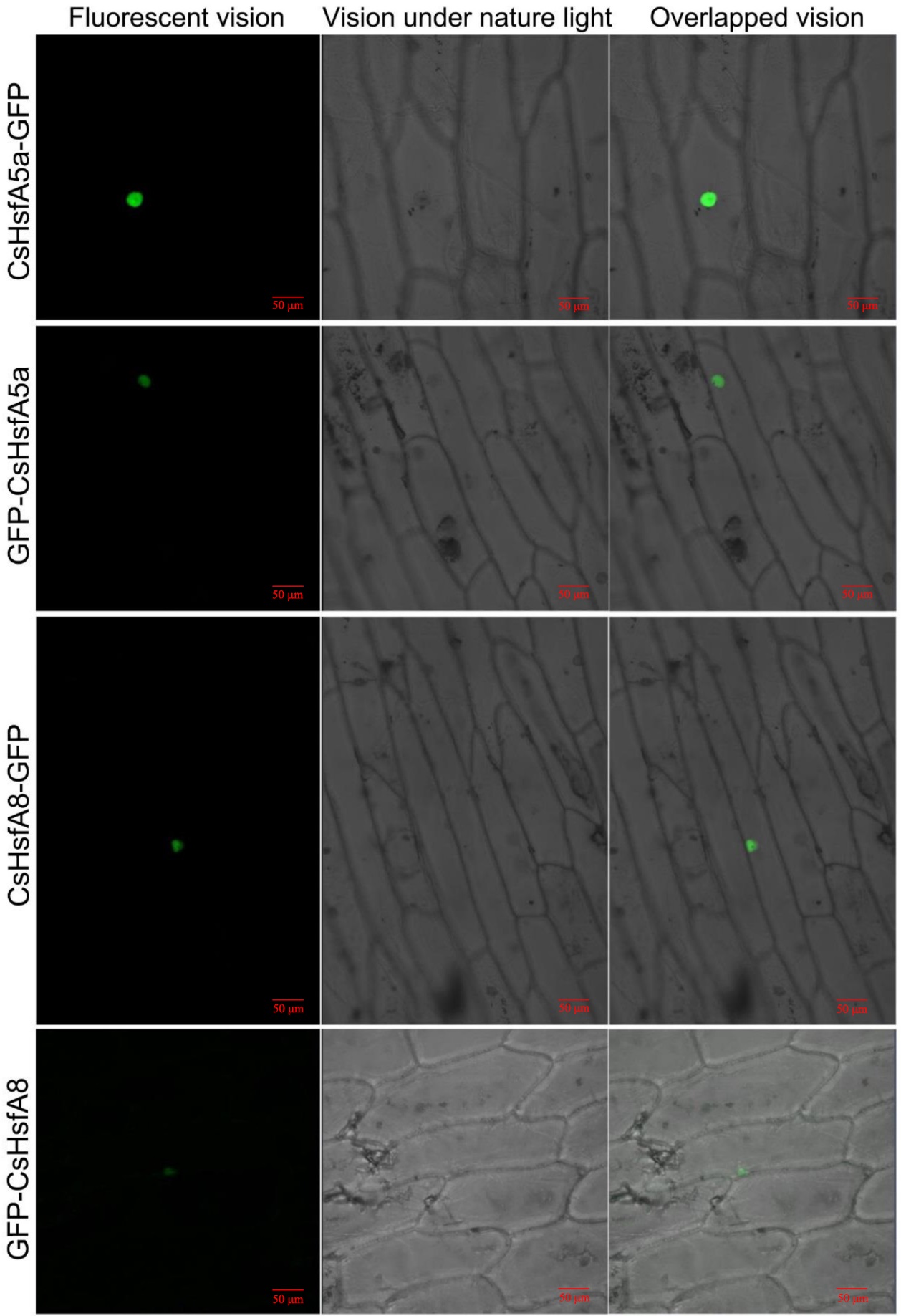
**

**Figure S2.** Subcellular localization of CsHsfA5a and CsHsfA8 in onion epidermal cells. CsHsfA5a-GFP and CsHsfA8-GFP indicate CsHsfA5a and CsHsfA8 fused with GFP at the C-terminus, and GFP-CsHsfA5a and GFP-CsHsfA8 indicate CsHsfA5a and CsHsfA8 fused with GFP at the N-terminus. The GFP signal was detected using a Zeiss LSM700 confocal laser-scanning microscope (Carl Zeiss Inc., USA) at 488 nm. Bars = 50 μm
